# Investigation of the Nanoparticle–Support Interaction Effects of Plasmonic Gold Nanoparticles Deposited on Nonpristine Aluminum and Steel Substrates

**DOI:** 10.1021/acsomega.5c11527

**Published:** 2026-09-23

**Authors:** Rosemary L. Calabro, F. John Burpo, Stephen F. Bartolucci, Joshua A. Maurer

**Affiliations:** † Department of Chemical and Biological Science and Engineering, United States Military Academy, West Point, New York 10996, United States; ‡ Photonics Research Center, United States Military Academy, West Point, New York 10996, United States; § U.S. Army Combat Capabilities Development Command-Armaments Center, Watervliet Arsenal, New York 12189, United States

## Abstract

Plasmonic nanoparticle (NP) sensors have been proposed to detect many analytes based on the principles of localized surface plasmon resonance (LSPR). They depend on spectral changes like peak wavelength, intensity, or broadness upon interaction with the analyte of interest, allowing optical readouts. However, they are also sensitive to factors like near-field coupling, size effects, and surface environment. For many applications, practical implementation of LSPR based sensors requires deposition of the NPs on a substrate. However, most studies to date have focused on highly crystalline or pristine materials which are impractical for scaled up industrial applications due to cost and size limitations. In this work, we investigate the NP–substrate interaction effects of gold NPs (AuNPs) deposited on two industrially relevant substrates: aluminum and steel. The AuNPs were dropcast on the substrates and the change in reflectivity before and after AuNP deposition was measured. Near-field coupling of the AuNPs was minimal after deposition on the substrates despite being observed on the AuNPs in solution. The spectral peak positions of the AuNPs were influenced by both the substrate and the surface environment. Variable angle measurements showed strong AuNP absorbance at low angles of incidence, with decreasing absorption as the angle increased. Larger particles had higher diffuse components while smaller particles favored specular reflection. Surface roughness showed limited influence on the overall observed intensities. From these observations, we outline a set of guidelines for solid supported plasmonic NP based sensors to optimize signal intensity and accuracy of measurement.

## Graphical abstract

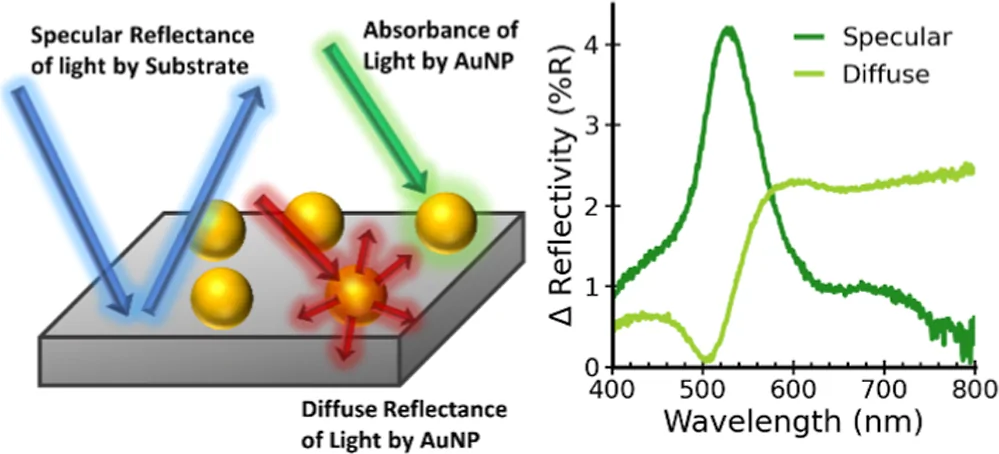

## 1 Introduction

Plasmonic nanoparticle (NP) sensors have been developed to detect a variety of analytes including gases,^1–3^ humidity and moisture,^4,5^ biomolecules,^6,7^ pH,^8,9^ environmental pollutants,^10,11^ pesticides^12^ and heavy metals.^13–15^ These sensors are composed of metal NPs and work based on the principles of localized surface plasmon resonance (LSPR). With LSPR, the electric component of incident electromagnetic radiation induces resonant oscillations of the conduction electrons in the NP, causing an amplification in absorption and scattering at the resonant wavelength.^16^ The LSPR properties are governed by the NP size, shape, and composition and are sensitive to changes in the surrounding dielectric environment. A change in the observed optical properties such as intensity, broadness, or peak wavelength is observed when the plasmonic sensor interacts with the analyte of interest.^17,18^ With direct plasmonic sensors, the NP itself undergoes a change upon interaction with the analyte. Examples of indirect sensors include Pd, Mg and Y that form metal hydrides,^1,17,19,20^ chemical reactions of silver NPs with H_2_S to form Ag_2_S shells,^21^ dissolution of NPs upon exposure to metal ions or pesticides allowing colorimetric readouts,^12,13^ and aggregation of NPs upon exposure to an analyte.^22,23^ With indirect sensors, NPs with good plasmonic properties, such as gold (AuNPs) or silver, rely on some change in the surrounding material that interacts with the analyte of interest. The analyte causes a change in the dielectric environment which shifts the LSPR properties. This has been demonstrated using core–shell particles,^17,24^ biofunctionalization,^6,7^ and coupling to dielectric nanostructures.^25^

One phenomenon that complicates the understanding of LSPR properties is the effect of near-field coupling. When particles are in close proximity to one another, they can couple together and form new resonant modes.^26^ This can provide interesting optical effects on a single particle level, and many studies have been done to probe these interactions. However, real-world application of the NPs requires faster throughputs, larger scales, nonpristine substrates, or irregular geometries and thus ensemble measurements are more practical. In an ensemble, near-field coupling can induce broadening and convolution of multiple LSPR interactions, which can be undesirable for sensing applications. Additionally, environmental effects, such as refractive index of the surrounding environment and contamination from solvents or gases, can cause shifts or changes in the LSPR.^17,27^ Finally, one major challenge toward implementation of plasmonic NPs is that many LSPR based measurements are done in solution.^12,13,22^ However, for many applications such as remote sensing, surface enhanced Raman scattering (SERS),^28^ and plasmon assisted catalysis,^29^ practical implementation of plasmonic NPs requires that NPs are deposited or immobilized on a substrate. This can lead to additional challenges due to NP—substrate interactions.

An understanding of the NP–substrate interaction in an ensemble is required, because the substrate properties can impact both the morphological arrangement and the dielectric environment of the NP. This will influence the LSPR properties and sensing capabilities of the NPs. For instance, when Au nanostructures were produced on a series of silicon-based ceramic substrates, the substrate environment greatly influenced the interparticle distance of the plasmonic NPs. This impacted both the observed peak wavelengths and the refractive index sensing sensitivity of the nanostructures.^30^ Many studies have investigated plasmonic NPs on substrates for sensing applications. However, the samples of interest typically featured pristine substrates and highly ordered, periodic nanoparticle structures and were prepared through complicated, time-consuming, or costly fabrication methods such as focused ion beam or electron beam lithography.^1,10,20,31^ While these systems provide valuable information into the overall mechanism of spectral response to an analyte, they present challenges in terms of scalability and practical implementation. Additionally, these studies typically use very pristine surfaces such as indium tin oxide, fused silica or atomically flat mirrors to host the NPs.^1,10,20,32^ However, for many applications, practical implementation of plasmonic NP sensors would require deposition on nonpristine, industrially relevant materials such as aluminum or steel. This reveals a need for a comprehensive set of design rules to generate plasmonic NP containing substrates for real-world sensors. The use of aluminum or steel substrates is expected to introduce NP–substrate interactions that influence the LSPR properties, and, thus, their response to an analyte of interest.

While metal NP–substrate interactions have received much attention in the past years for systems such as NPs on a mirror,^33,34^ on transition metal dichalcogenides,^35^ or on sapphire,^36,37^ the substrates are typically crystalline pristine materials, unlike steel or aluminum. Thus, in order to implement the simpler, nonpristine, easier to obtain, and less costly aluminum and steel substrates into optical applications, there is a requirement for empirical studies that investigate what impact they have on the optical properties of plasmonic NPs. Systems containing plasmonic NPs on steel and aluminum oxide have been studied in the context of catalysis^38^ and electrocatalysis,^39–42^ but these studies did not consider the changes in optical properties. Therefore, we aimed to fill this knowledge gap by empirically examining the spectral properties of various types of AuNPs deposited on two industrially relevant nonpristine substrates: aluminum and steel. We show that separating out the specular and diffuse components of reflectance measurements can lead to different observed peak wavelengths. Additionally, we show the importance of measurement angle on signal intensity. We then demonstrate how the stabilizing environment of the AuNPs influences their interaction with the substrates and discuss how other parameters such as AuNP shape and size and substrate roughness impact the spectral properties. Finally, we use these results to outline a set of guidelines for designing plasmonic NP-based sensors.

## 2 Experimental Section

### 2.1 Materials

All chemicals were used without further purification unless otherwise specified. 50 nm AuNPs in phosphate buffered saline, 50 nm gold nanourchins, and 100 nm gold nanourchins were purchased from Strem Chemicals. 50 nm cubic gold nanoparticles, 50 nm cetyltrimethylammonium bromide (CTAB) stabilized gold nanoparticles, and 50 nm citrate stabilized gold nanoparticles were obtained from Nanopartz. 1-Hexadecanethiol (>97.0%) and 1-propanethiol (>98.0%) were purchased from TCI America. Isopropyl alcohol (BioReagent, ≥99.5%), and acetone (ACS reagent ≥ 99.5%) were purchased from Sigma-Aldrich. Poly­(methyl methacrylate) (PMMA) beads, average M.W. 35,000 was purchased from Acros Organics. The 9 μm DiaPro solution, 3 μm DiaPro solution, and OP-S NonDry colloidal silica suspension used for polishing were purchased from Struers. Steel (4340 steel) and aluminum (7079 aluminum) substrates were cut and polished prior to being used for analysis.

### 2.2 Characterization

Profilometry measurements were collected with a KEYENCE VR-3000 wide-area 3D measurement system. Solution phase ultraviolet–visible spectroscopy measurements were collected on a Jasco V-630 spectrophotometer. True absorbance and reflectometry measurements of the AuNPs on solid substrates were collected using an Agilent Cary 7000 Universal Measurement Spectrophotometer with a UV–vis–NIR Diffuse Reflectance Accessory (integrating sphere). Scanning electron microscopy (SEM) images were measured on a JEOL JSM-IT500HR field emission SEM and scanning transmission electron microscopy (STEM) images were collected with a FEI Helios Nanolab 600i Dual Beam FIB-SEM. Dynamic light scattering measurements were obtained with a Malvern Panalytical Zetasizer Ultra equipped with a Red 10 mW HeNE 632.8 nm laser.

### 2.3 Substrate Polishing

The steel and aluminum substrates were polished using a Struers LaboSystem. The steel substrates were polished with 600 grit, 9 μm, 3 μm and then 1 μm polishing pads to achieve the final polish. The 9 and 3 μm polishing pads used DiaPro solutions and the 1 μm polishing pad used the OP-S NonDry colloidal silica suspension for polishing. For the steel substrates, the medium level polishing was achieved by ending with the 9 μm polishing step and the rough level of polishing was achieved by only using the 600 grit polishing pad. The aluminum substrates were polished using 400 grit, 600 grit, 9 μm, and 3 μm polishing pads. For the aluminum substrates, the medium level polishing was achieved by ending with the 600 grit polishing step and the rough level of polishing was achieved by only doing the 400 grit polishing step.

### 2.4 Reflectometry Measurements

The reflectometry measurements were obtained using an integrating sphere that allowed both rear and center mounting of the substrates. The center mount configuration was only used for variable angle measurements and all other reflectometry measures were collected using the rear mount configuration. For a typical measurement, the reflectometry spectra of the bare substrates were measured. Then, the AuNPs were centrifuged at 5000*g* for 10 min using a VWR High Speed Microcentrifuge and were redispersed in isopropyl alcohol (IPA) using 1/3 of the original volume. The AuNPs were then dropcast on the substrates by dropping 5 μL of the washed solution in the center of the substrates and drying with a heat gun. This was repeated for a total of 20 drops. For a typical deposition, the AuNPs covered an area of approximately 3–4 cm^2^. Finally, the reflectometry spectra of the substrates with the dropcast AuNPs were measured and compared to the bare substrates. For rear-mounted measurements a 1.2 cm^2^ circular area of the substrate was placed facing the inside of the integrating sphere. Incident light interacted with the substrate at an 8° angle relative to the normal of the substrate. A specular exclusion port located at 8° in the opposite direction of the incident light could be opened to separate out the specular component and include only the diffuse component of reflected light in the measurement. For variable angle measurements, the substrates were measured from 5° to 85° in increments of 10° and all measurements contained both the specular and diffuse component of the reflected light.

#### 2.4.1 Ligand Exchange

To coat the AuNPs with the thiol ligands, a simple ligand exchange process was done. First 3 mL of the citrate stabilized AuNPs were centrifuged for 10 min and redispersed in IPA. Then 3 μL of 1-propanethiol or 10 μL of 1-hexadecanethiol was added to the AuNPs. The solution was stirred for 1 h. The AuNPs were then centrifuged again and redispersed in IPA.

## 3 Results

Figure 1a shows a representative, normalized absorbance spectra of three types of 50 nm AuNPs with different stabilizing environments that were dispersed in water. All three AuNPs had peaks centered in the 527–533 nm range with slightly different peak full width half maximums (fwhm). The citrate-coated particles had the narrowest peak, with a slightly broader peak for the phosphate-stabilized particles and the CTAB particles having the broadest peak. These changes could originate either from inhomogeneous broadening due to the samples having subtly different size dispersions or due to the different stabilizing environment causing slightly different dielectric environments, which could affect both the peak position and the fwhm.^27^ When the samples were washed and redispersed in IPA, two changes in the spectra occurred. First, the peaks originally centered in the 527–533 nm range red-shifted slightly and broadened, which is explained by the different indices of refractions for water and IPA influencing the LSPR peak positions and fwhm.^27^ Second, a very broad peak appeared in the 650–750 nm region for all three samples. This is likely due to some AuNP agglomeration occurring in the IPA, which causes near-field coupling of the particles and allows a broad and red peak to appear.^26,43,44^ Dynamic light scattering measurements (Figure S1 and Table S1) confirm that the AuNPs tend to agglomerate when transferred from water to IPA, as indicated by the increase in the measured polydispersity indices, peak means by intensity, and the appearance of second peaks for some samples dispersed in IPA. As anticipated, the spectral intensity at long wavelengths and the nanoparticle agglomeration observed in DLS is most pronounced with the CTAB coated particles due to the interactions of the hydrophobic surface ligand.

**Figure 1:**
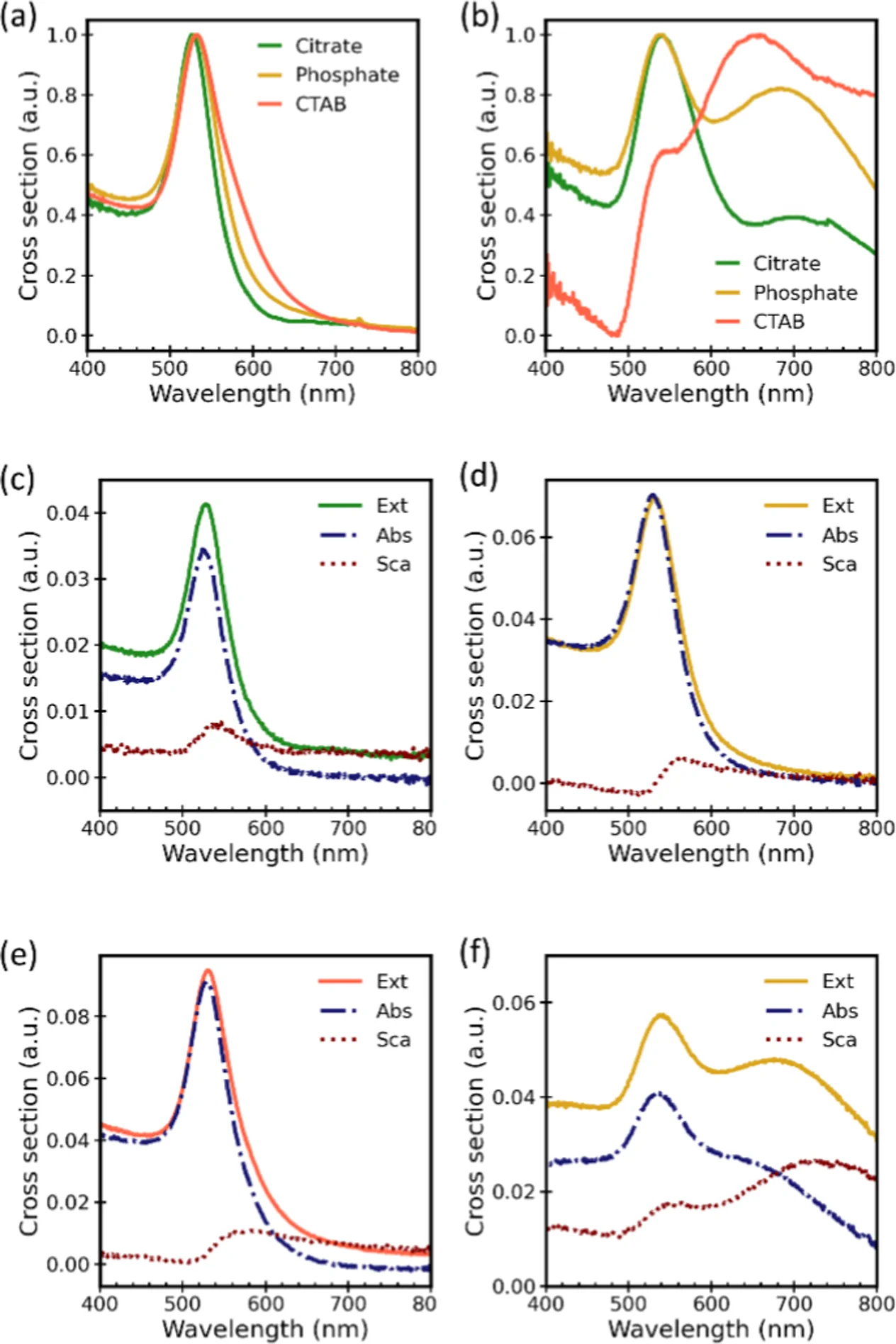
Solution phase spectra of the various 50 nm AuNPs dispersed in water (a), and IPA (b), and true absorbance measurements (c–f) show the extinction (Ext), absorbance (Abs) and scattering (Sca) components of the 50 nm AuNPs dispersed in water and stabilized by citrate (c), phosphate buffered saline (d), CTAB (e), and the phosphate buffered saline AuNPs dispersed in IPA (f).

True absorbance measurements were collected for the samples in water (Figure 1c–e and Table S2) using an integrating sphere according to a procedure described previously.^45–47^ The overall extinction was collected with the sample placed in front of the integrating sphere, which allows only transmitted light to reach the detector. To collect the true absorbance, the sample was then moved to the center of the sphere which allows both transmitted and scattered light to reach the detector. The scattering component was then calculated by subtracting the true absorbance spectra from the overall extinction spectra.^45–47^ For all three samples, the main contribution to the overall extinction was absorbance with a low-intensity scattering component that was red-shifted relative to the absorbance, as is consistent with Mie theory.^16^ When the AuNPs are transferred to IPA (Figure 1f), it is apparent that the main peak originating from LSPR of individual AuNPs in solution is still predominantly absorbance. However, the redder peak that originated from near-field coupling has a much higher scattering component, which is often observed in nanoparticle agglomerates.^26,43,44^

The AuNPs were then deposited on steel and aluminum substrates using a simple dropcast method. The AuNPs were deposited on the metal substrates from solutions dispersed in IPA due to the higher volatility of IPA compared to water. SEM images of the phosphate stabilized AuNPs (Figure 2a,b) show that the particles tend to dry on the substrates, with some individual particles isolated from others, but most particles arranged in contact with other AuNPs. In some cases, only a few particles are in contact, but in others the particles appear to be part of much larger aggregates. Cluster analysis of the AuNPs was done using ImageJ^48^ and is described in detail in the Supporting Information (Figure S2). It was determined that for both the steel and aluminum substrates, over 80% of the phosphate stabilized AuNPs were in contact with at least one other particle. A majority of the clusters contained less than 5 AuNPs, but there were still larger ones present; however, most were under 25 AuNPs (Figure 2c–e). Cluster analysis was done on the citrate and CTAB coated particles as well and showed similar results (Figure S3).

**Figure 2:**
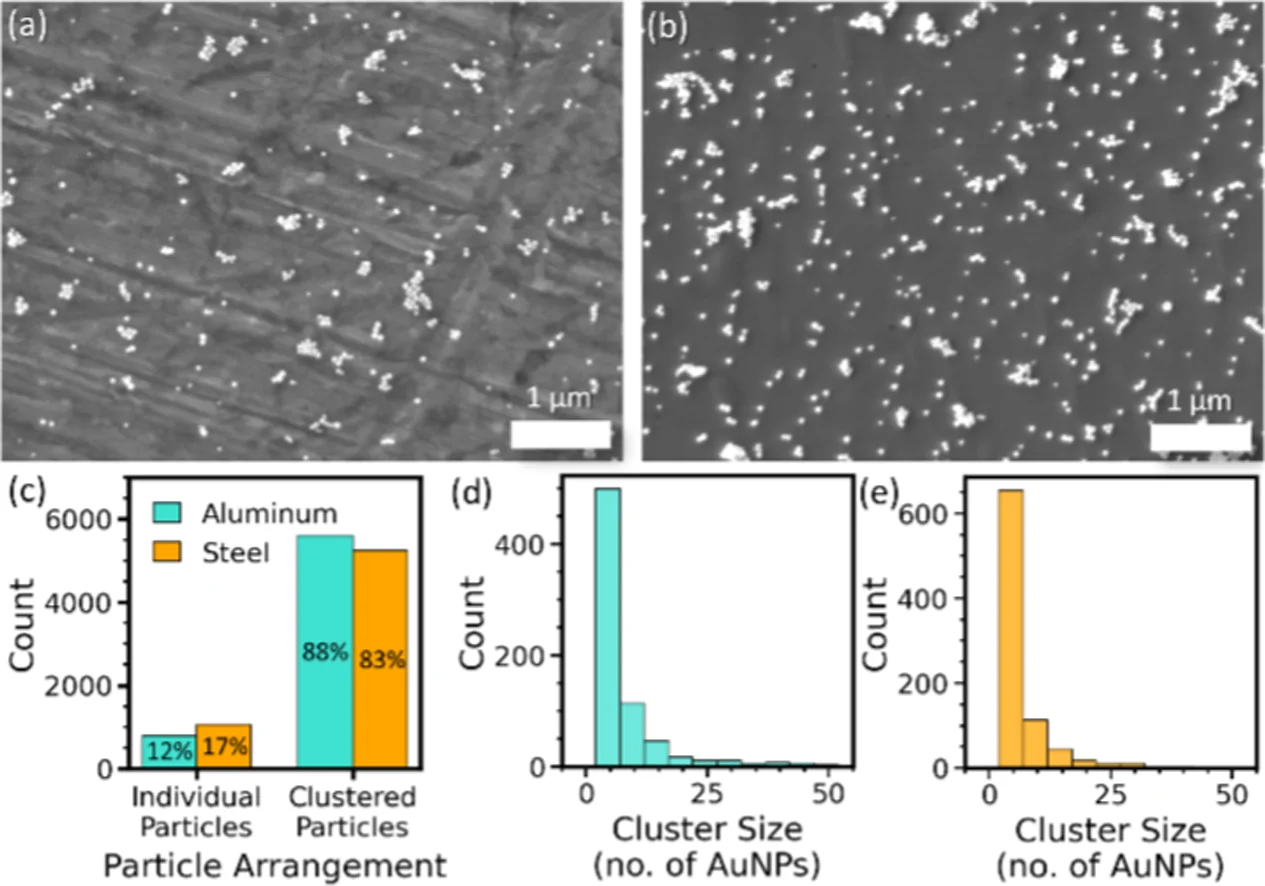
SEM images of the phosphate stabilized AuNPs deposited on aluminum (a), and steel (b) substrates, a box plot summarizing the cluster analysis (c), and histograms of the aluminum (d), and steel (e) cluster sizes.

Comparison of the solution phase spectra with the reflectivity spectra allows insight into how the optical properties change when deposited on substrates. The reflectivity measurement approach allowed for collection of spatially averaged measurements of the AuNPs deposited on the two substrates. The reflectivity (% *R*) of the substrates was measured using an integrating sphere both before and after dropcasting the AuNPs on them. The incident light interacted with the substrates at an 8° angle relative to the normal of the substrate, and a specular exclusion port that was located at 8° in the opposite direction of the incident light could be opened to remove the specular component of the reflected light, leaving only the diffuse component, or it could be closed to allow inclusion of both the specular and diffuse components in the measured spectra (Figures 3a,f and S4–S7). Figure 3b shows the reflectivity of the polished aluminum substrate both with and without the citrate coated AuNPs dropcast on it. There is a clear decrease in the reflectivity in the wavelength region where the solution phase AuNPs had an absorbance maxima. This decrease in reflectivity is then attributed to the absorbance of the AuNPs causing less light to be reflected, and an adjusted spectra, which shows the change in reflectivity (Δ% *R*), can be plotted by subtracting the AuNP measurement from the bare substrate and then offsetting the spectra so that the minimum intensity is zero (Figure 3c).

1 $$\Delta \%\;R={\%\;R}_{substrate,specular}-{\%\;R}_{AuNP,specular}$$

**Figure 3:**
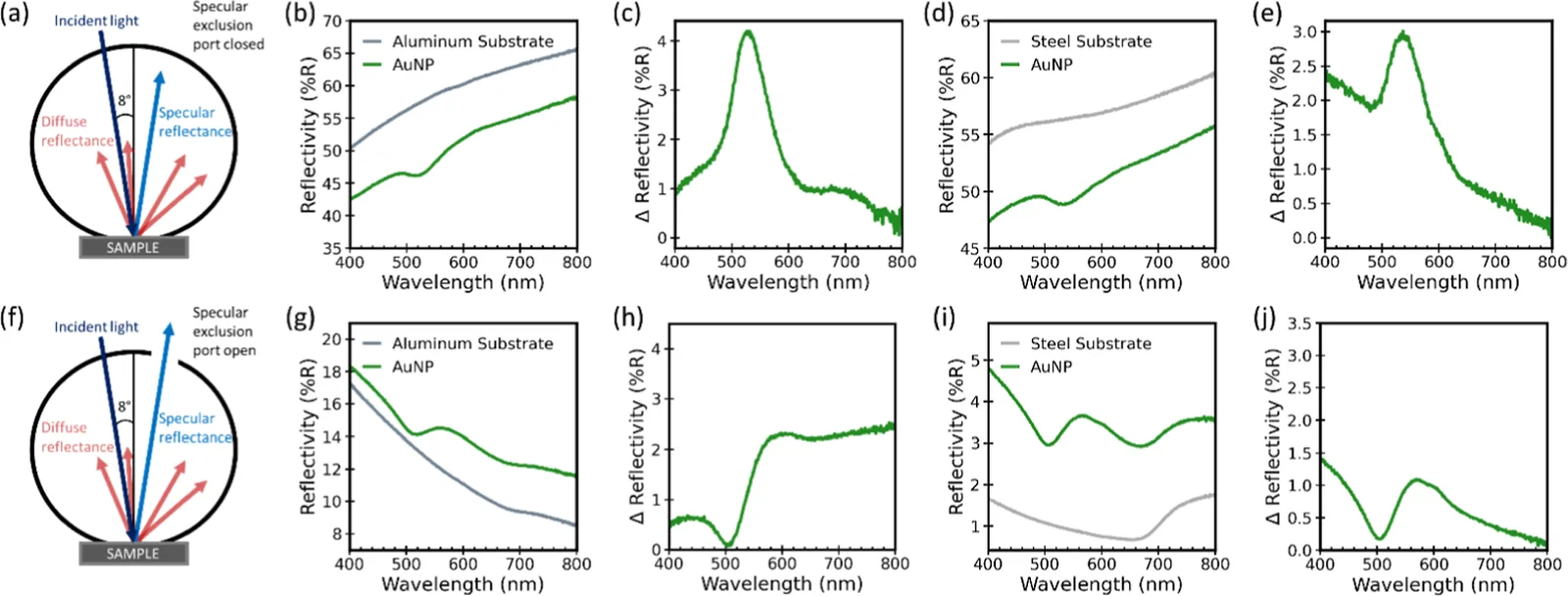
Specular and diffuse raw and adjusted reflectivity (% *R*) spectra of citrate stabilized AuNPs on various substrates. When the integrating sphere specular exclusion port was closed (a), the % *R* included both specular and diffuse components and absorbance of the AuNPs resulted in a decrease in % *R* relative to the blank substrate as seen on the specular spectra for AuNPs on aluminum raw (b) and adjusted (c), and AuNPs on steel raw (d) and adjusted (e). When the specular exclusion port was open (f), the % *R* only included the diffuse component and any diffuse reflectance resulted in an increase in % *R* as shown on the diffuse spectra for AuNPs on aluminum raw (g) and adjusted (h), and AuNPs on steel raw (i) and adjusted (j).

This offset is done due to a slight loss in intensity from impurities in the IPA (Figure S8). The same effect is observed for the AuNPs deposited on the steel substrates (Figure 3d,e). When the specular component is excluded, a different effect is observed. The bare substrate has a much lower % *R* which suggests that the majority of reflected light by the substrate is due to specular reflectance. Once the AuNPs are deposited on the substrates, an increase in the % *R* is observed with peaks in similar wavelength regions to the solution phase AuNPs. However, these peaks are slightly red-shifted relative to the spectra that include the specular component. The diffuse reflectance is collected at all angles within the integrating sphere and can be considered similar to scattering effects of the AuNPs. This is in agreement with the true absorbance measurements, which showed the scattering component was redder than the absorbance component of the overall extinction. Scattering effects would also cause an overall increase in the observed reflectivity, because they allow more light to reach the detector, unlike absorbance which causes an overall decrease. For these reasons, the adjusted spectra for diffuse measurements are calculated by subtracting the bare substrate from the measurement with the AuNPs (Figure 3g–j).

2 $$\Delta \%\;R={\%\;R}_{AuNP,diffuse}-{\%\;R}_{substrate,diffuse}$$

The calculation of the adjusted spectra is discussed in further detail in the Supporting Information along with the raw spectra for all remaining reflectivity spectra. For any following reported reflectivity measurements, the adjusted spectra are presented in the main text and the raw data is available in the Supporting Information. When the spectrum includes the specular component, it is referred to as “specular” and when it excludes the specular component it is labeled as “diffuse”.

The AuNPs that were stabilized by CTAB and phosphate buffered saline showed similar effects for both the specular-included and diffuse-only measurements (Figures S6 and S7), however, the CTAB stabilized AuNPs had broader and less defined peaks. For all three types of AuNPs, the specular component had much higher intensities, with only weak intensities from the diffuse components. The diffuse components were also broader and less defined, with their higher-intensity regions extending toward the near-infrared (NIR). The peak positions for all three types of 50 nm AuNPs are summarized in Table 1. In general, the specular peaks for the samples deposited on steel had red-shifted peaks relative to the samples deposited on aluminum, which were bluer. While both substrates showed broadening in the spectral features relative to their solution phase counterparts (Table S3), the aluminum substrates had narrower peaks when compared to the steel substrates. The citrate and phosphate stabilized AuNPs deposited on aluminum had peaks very close to the solution phase peak position, but the CTAB sample was red-shifted by 25 nm. The diffuse component was red-shifted relative to the specular component and in some cases the diffuse spectra was too broad to estimate a peak wavelength.

**Table 1:** Spectral Peak Positions of Various AuNPs Measured in Solution and on Aluminum and Steel Substrates

sample	solution phase AuNPs (nm)	ext (nm)	abs (nm)	sca (nm)	AuNPs on aluminum specular (nm)	AuNPs on aluminum diffuse (nm)	AuNPs on steel specular (nm)	AuNPs on steel diffuse (nm)
citrate AuNPs	527	528	524	542	528	606	537	570
CTAB AuNPs	533	530	524	588	558	701	560	680
phosphate AuNPs	531	532	530	567	528	broad features—λ_max_ > 750	541	broad features—λ_max_ > 750
Au cubes	539	539	536	547	546	broad features—λ_max_ > 750	544	broad features—λ_max_ > 750
50 nm urchins	561	559	556	580	566	685	560	673
100 nm urchins	655	652	618	669	605	670	600	641

Assuming there are similar NP–NP interactions on both substrates, these results suggest that the citrate stabilized and phosphate buffered AuNPs had a stronger NP–support interaction with the steel substrate compared to aluminum. This is best explained by the fact that the aluminum readily forms a thin oxide layer on the surface, which could help protect the substrate from coupling to the AuNPs.^49,50^ Near-field coupling has also been reported for AuNPs deposited on other substrates, such as indium tin oxide, with controllable peak wavelengths as a function of cluster size.^32^ However, we do not observe a similar effect for the AuNPs deposited on aluminum and steel regardless of the AuNP stabilizing environment, despite the fact that this was observed in the solution phase spectra when dispersed in IPA (Figure 1b). Additionally, if near-field coupling was strongly influencing the spectral properties, we would expect our samples to have very broad peaks due to inhomogeneous broadening from the nonuniform cluster sizes we observed. While we do not observe significant broadening in the citrate stabilized or phosphate buffered AuNPs, we do observe that the CTAB coated AuNPs had much broader spectra with less defined peaks. The DLS indicated that the CTAB coated particles had higher polydispersity indices and peak mean intensities in both water and IPA (Table S1) compared to the other particles. This suggests that the CTAB particles could have a higher degree of agglomeration or particle–particle interaction and could account for the broader and red-shifted spectral features when deposited on the steel and aluminum substrates. Finally, reproducibility tests (Figure S9) show that similarly prepared AuNPs on substrates have similar spectral shapes. The most notable difference is the overall intensity which is attributed to the nonuniformity of the NP arrangement due to the drop casting technique. Lower intensity measurements also yielded broader peaks (Table S4), so in general it is preferred to prepare substrate with a higher number of particles to improve signal. These results show that the stabilizing environment influences whether the AuNPs interact more strongly with the substrates or other particles. For some stabilizing environments the AuNP–substrate interactions play a stronger role than any interactions between the AuNPs. For other systems, the stabilizing environments may favor particle–particle interactions. This emphasizes the importance in selecting an appropriate stabilizing environment for the desired application. These results are also promising for sensing applications because with certain stabilizing environments, such as citrate or phosphate buffered saline, the metal substrates can help eliminate coupling effects that could influence the sensor’s performance.

Variable angle measurements of the AuNPs on steel and aluminum were collected, since it has been shown in other systems that the measurement angle influences the intensity of reflectivity for plasmonic NPs.^1^ Figure 4 shows the adjusted variable angle measurements for the citrate stabilized AuNPs on both aluminum and steel. All samples were measured from 5° to 85° relative to the normal of the substrate in increments of 10° and the measurements included both the specular and diffuse components of the reflected light. The adjusted spectra were offset, so that the Δ% *R* at 800 nm was equal to zero because the particles did not absorb or scatter at that wavelength. Scatter plots of the peak wavelength at each measurement angle relative to the lowest observed intensity are shown in Figure 4c,d. For both substrates, the maximum Δ% *R* was observed at 5°. As the angle increased, the overall Δ% *R* decreased with minimal peak features observed at angles greater that 45°. This same effect was observed for the CTAB and phosphate buffered saline AuNPs (Figures S10 and S11). Based on these results, it is apparent that the measurement angle plays a significant role in terms of signal output, with low angles favoring a better signal readout. These results provide insight on how to obtain the optimum signal when designing LSPR based sensors, plasmon-assisted catalysts, or SERS devices. The best plasmon interaction or signal would be acquired at low angles relative to the normal of the substrate. Additionally, this indicates that for measurements that rely on a change in the LSPR peak intensity, control of the angle should be of high priority since wide angle measurements can lead to low observed intensities. Finally, since it is not possible to separate out specular and diffuse components during variable angle measurements, particles that only have a strong specular component will allow readouts that are straightforward and unconvoluted.

**Figure 4:**
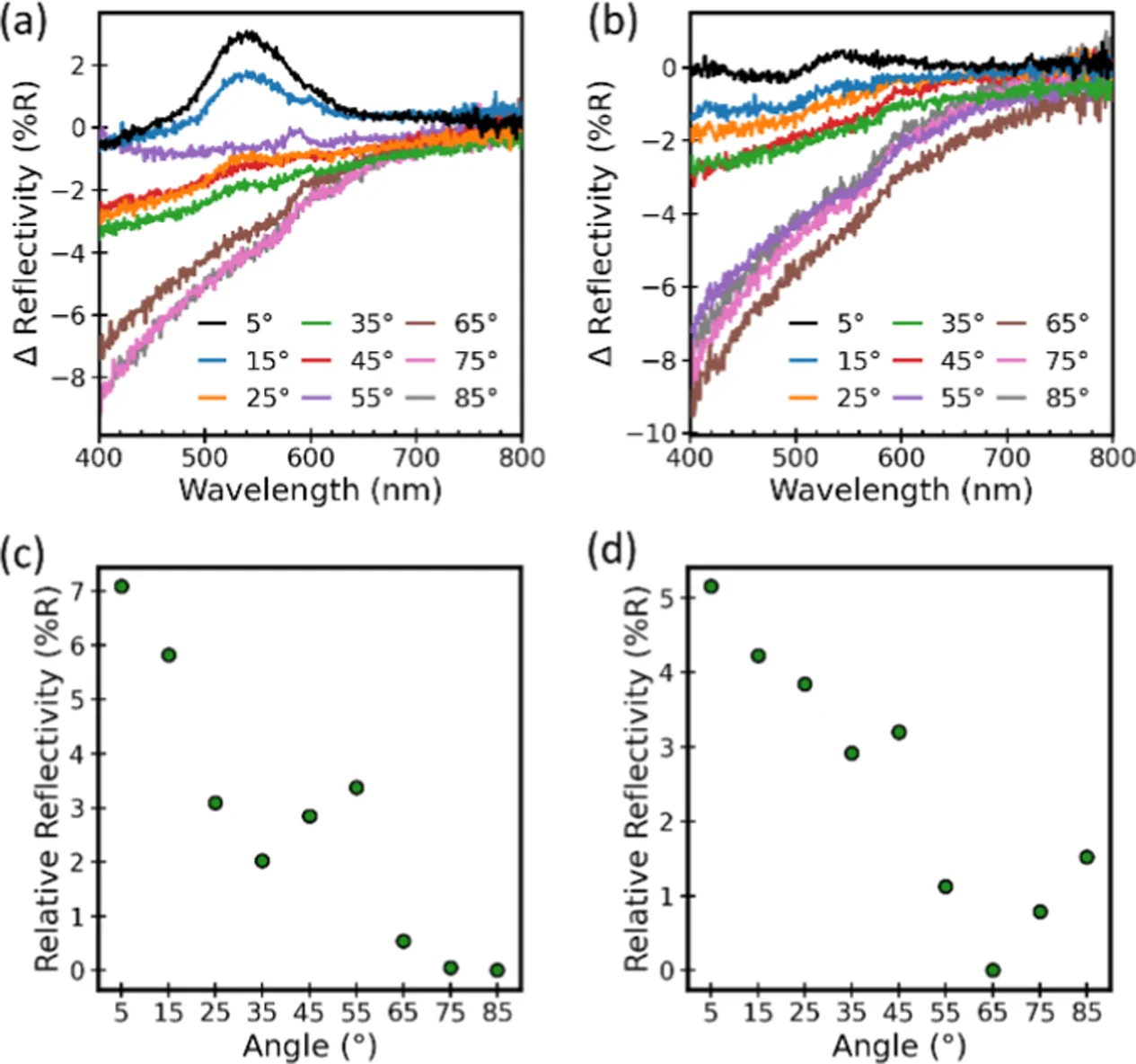
Variable angle measurements of the citrate stabilized AuNPs deposited on aluminum (a) and steel (b) and plots of the relative Δ% *R* at their peak wavelength at various measurement angles for the AuNPs deposited on aluminum (c) and steel (d).

The influence of the size and shape of the AuNPs was also considered (Figures 5 and S12 and S13). Representative spectra for the solution phase 50 nm Au cubes, 50 nm Au urchins, and 100 nm Au urchins show similar properties to the spherical AuNPs, as shown in Figure 5. The 50 nm urchins had a slightly redder and broader peak compared to the 50 nm cubes; however, for both samples, the main extinction mechanism was absorbance with only a weak scattering contribution. Similarly, when considering their adjusted reflectivity spectra, the specular component, which is due to absorption, was much stronger and better defined than the diffuse spectra, which had broad and weak features that extended toward the NIR. Similar to the case of the spherical AuNPs, the steel substrates yielded a higher degree of spectral broadening than the aluminum substrates (Table S3). Additionally, the specular peak positions were similar for both the aluminum and steel substrates and did not have the 10+ nm shifts from the solution phase NPs that were observed for the spherical AuNPs deposited on steel. This could be due to less substrate–particle interaction for the cubes and urchins compared to the spheres. The 100 nm urchins had a much redder peak wavelength compared to the 50 nm urchins, which is expected due to their larger size. Their true absorbance measurements show that they have a much higher contribution from scattering compared to the other samples (Figure 5d). This is consistent with Mie theory, which predicts larger scattering components for larger sized NPs.^16,45^ When looking at their reflectivity spectra (Figure 5e–j), they have significantly broader specular reflectance. Additionally, the peak positions of their specular component are much bluer than that observed for the solution phase particles. However, this was also the case for their true absorbance peaks. When looking at the diffuse components, the 100 nm Au urchins have broad peaks at 670 nm for the AuNPs on aluminum and 641 nm for the AuNPs on steel (Figure 5i–j). On aluminum, the peak matches well with the observed scattering component from the true absorbance measurement. On steel the peak is blue-shifted, which could be due to the stronger interaction with the steel substrates. Nevertheless, these results show that samples with larger scattering components will also have large diffuse components. This could be useful for some applications, however, it also could complicate the interpretation of optical readouts during sensing applications, so the particle size should be carefully considered when choosing NPs to use for plasmonic sensors.

**Figure 5:**
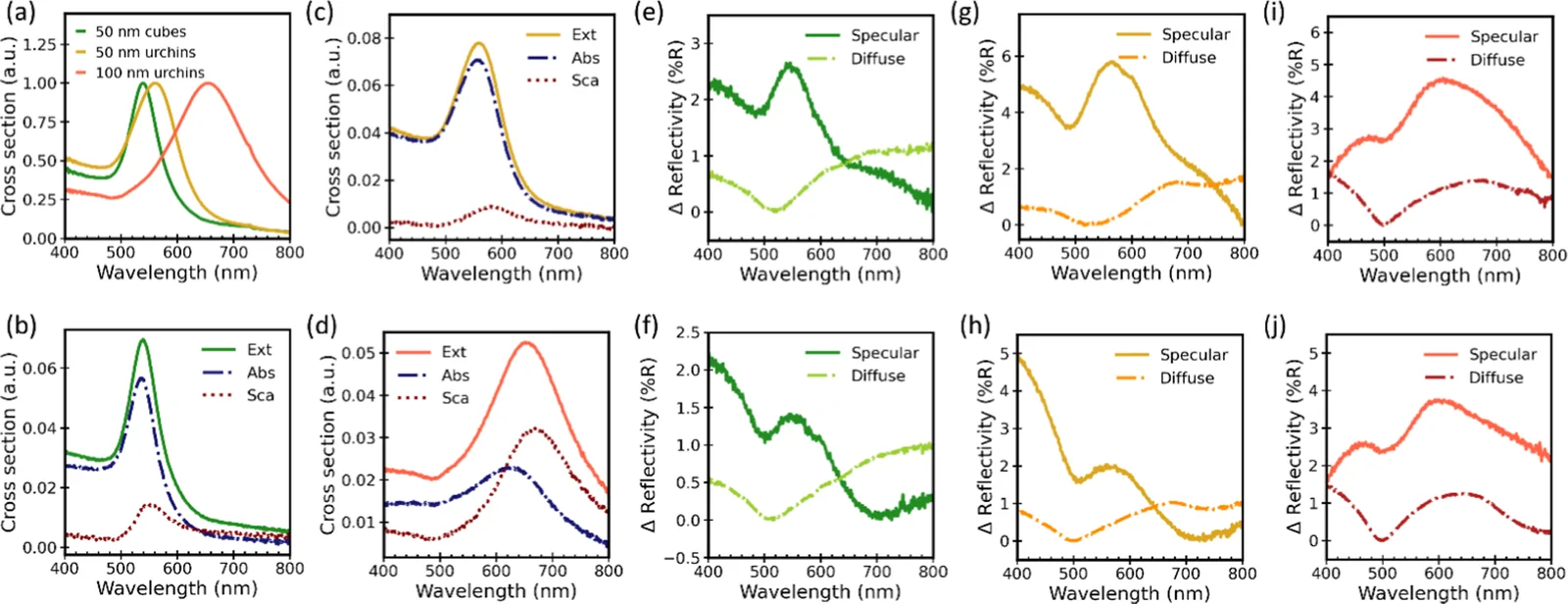
Spectral measurements of various Au shapes. The solution phase spectra of the various shapes dispersed in water (a), true absorbance measurements of the cubes (b), 50 nm nanourchins (c), and 100 nm nanourchins (d), and specular and diffuse reflectivity measurements of the shapes deposited on substrates including cubes on aluminum (e) and steel (f), 50 nm urchins on aluminum (g) and steel (h), and 100 nm urchins on aluminum (i) and steel (j).

The effects of surface environment was studied in further detail through a thiol ligand exchange and by coating the substrates with a polymer layer. For the ligand exchange, two thiol ligands of various lengths replaced the citrate capping agent that stabilized the AuNPs. The citrate stabilized AuNPs were first washed and redispersed in IPA which allowed solubility of the thiol ligands. As previously discussed, this resulted in a red shift of the LSPR and the appearance of an additional peak due to near field coupling (Figure 6a). After the ligand exchange, the LSPR of the thiol-coated AuNPs shifted back toward the blue with the shorter 1-propanethiol (C_3_–SH) coated AuNPs having the shortest wavelength, and the longer 1-hexadecanethiol (C_16_–SH) coated AuNPs having an intermediate peak. The surrounding solvent and capping agents will influence the dielectric environment, which shifts the LSPR.^17^ Changing the solvent from water to IPA clearly had major impact on the citrate stabilized AuNPs, but the influence from the thiol ligands seemed to dominate over solvent effects following the ligand exchange. The additional peak from near field coupling also blue-shifted as the ligand length increased, suggesting that the longer thiols provide improved solubility in IPA. The ligand effects are also apparent after depositing the AuNPs on the aluminum and steel. Here, the citrate particles had the bluest peak. Ligand exchange with the C_3_–SH shifted the observed peaks toward the red, and the peak position was shifted even further with the longer C_16_–SH ligand. The citrate particles on aluminum had a peak that was very similar to the citrate AuNPs in water, however, the steel peak position was red-shifted by 9 nm. This red shift was less prominent in the thiol coated AuNPs. Again, the peak positions of AuNPs on aluminum were within 1–2 nm of the solution phase spectra, however, when depositing on steel there was a 4 and 5 nm red shift for the C_3_–SH and C_16_–SH coated AuNPs, respectively. These findings show that the surface coating of the AuNPs can be tuned to better protect the particles from interactions with the substrates, especially the steel. This can allow the spectral properties to more closely match what is observed for the AuNPs in solution. Surface coatings of the AuNPs could then be used as a strategy to design sensors that respond only to the analyte of interest, with minimal influence from the substrate.

**Figure 6:**
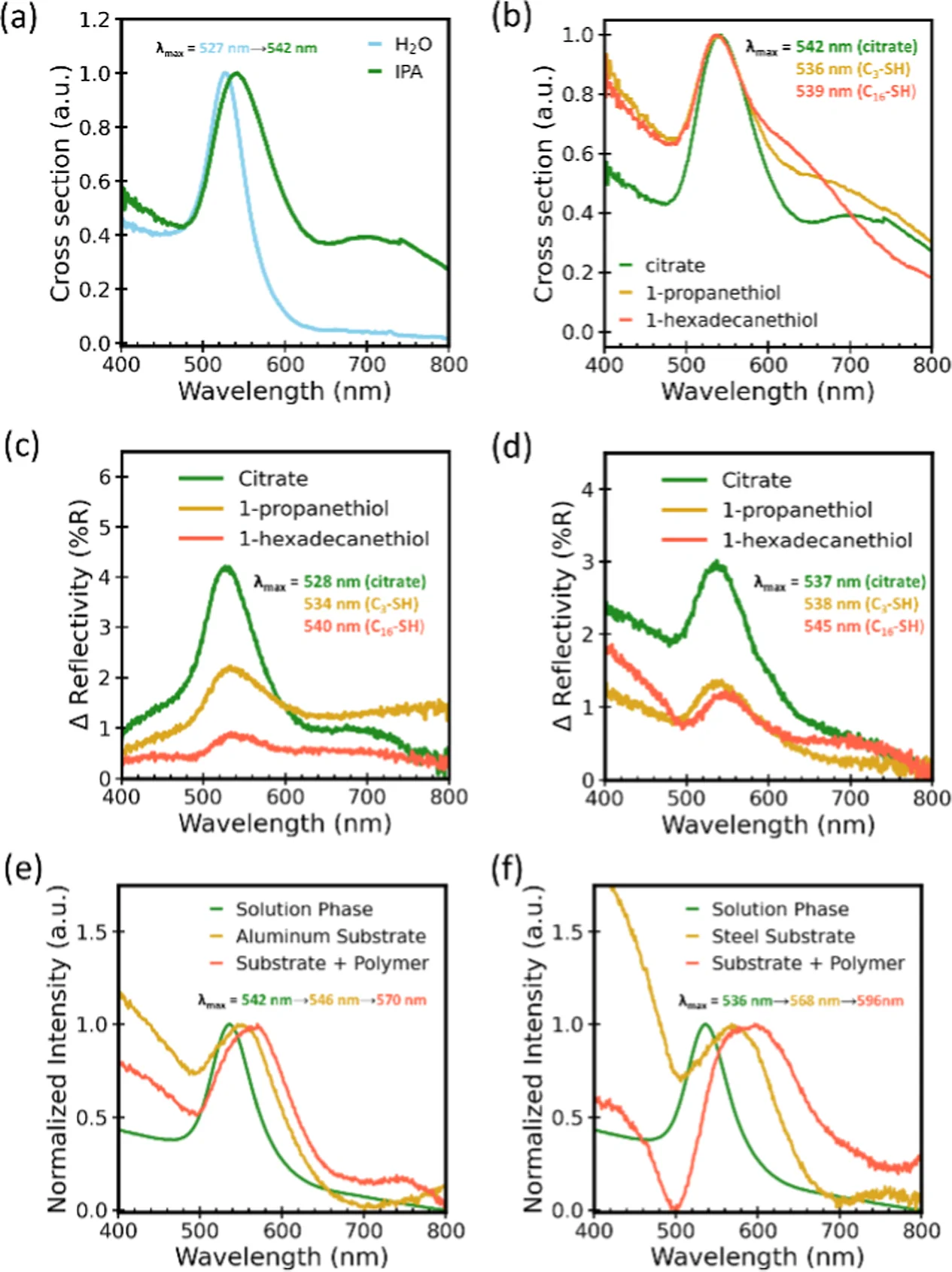
Solution phase spectra of the citrate stabilized AuNPs dispersed in water and IPA (a) after the thiol ligand exchange (b), and reflectivity measurements after depositing the thiol coated AuNPs on aluminum (c) and steel (d) substrates. Substrate measurements of citrate stabilized AuNPs before and after deposition of a PMMA polymer layer on aluminum (e) and steel (f) substrates.

The influence of the surrounding environment was further examined by introducing a polymer coating to the substrate after AuNP deposition. Citrate coated AuNPs were first drop cast on steel and aluminum substrates and measured. Then, 100 μL of a 20 mg/mL solution of 35 kDa poly­(methyl methacrylate) (PMMA) dissolved in acetone was dropcast on the substrates and allowed to dry. Figure 6e,f presents the normalized spectra of the solution phase AuNPs, and the substrates both before and after PMMA deposition. Introduction of the PMMA increases the refractive index of the surrounding environment of the AuNPs, since PMMA has a higher refractive index than air.^51^ Increasing the refractive index in known to cause a red shift in the LSPR of nanomaterials^52^ which is the trend we observed on both the aluminum and steel substrates. In both cases, the peak positions red-shifted by over 20 nm. However, the broadness for the aluminum substrate was similar both before and after PMMA deposition, while the broadness increased for the steel substrate by 16 nm. These results confirm that the plasmonic properties of AuNPs on aluminum and steel substrates undergo the expected red shift when increasing the refractive index of the surrounding media. Since many sensing applications rely on a shift in refractive index from interaction with an analyte, this further supports the possibility of using aluminum and steel as nanoparticle substrates.

Finally, the impact of substrate surface roughness was considered (Figures S15–S17 and Table S5). For many nanoparticle–substrate studies, atomically precise or mirror finish substrate was used, however they are costly to make and difficult to scale up. In this study, nonpristine surfaces were specifically selected because they are more likely to be encountered in industry. While the steel and aluminum substrates were polished prior to measurement, their arithmetic mean roughness values (Ra > 0.3 μm) were all much higher than the average nanoparticle diameters of approximately 50 nm. Even with valley–peak heights on the substrates that are much higher than the individual nanoparticle sizes, we still can obtain spectra with well-defined peaks. These results show that the surface roughness, and thus the degree of polishing of the substrates, does not play a prominent role in the AuNP–substrate interactions. However, the AuNPs deposited on the aluminum substrates had better defined peaks, regardless of degree of surface roughness, while the AuNPs deposited on steel had broader, less defined peaks, especially the CTAB and phosphate stabilized particles. These observations suggest that the surface roughness had minimal impact on the overall measurements, but that the type of substrate and surrounding environment plays a much more prominent role in the spectral properties.

## 4 Discussion

The results shown previously indicate how different types of measurement conditions yield different reflectometry responses. Different factors such as the particle size, stabilizing environment, angle of measurement, and substrate roughness all had varying degrees of influence on the overall spectral intensities, peak wavelengths, and broadness. Many of these results can be generalized to other types of plasmonic particles. Based on these observations, we propose an empirical guideline of parameters to consider when designing LSPR based sensors which are summarized as a set of design rules in Table 2.

**Table 2:** Empirical Design Rules for Nanoparticle–Substrate Based Plasmonic Sensors

design parameter	corresponding figure (s)/table (s)	guidelines
1. particle choice	Figures 5, S6, S7 and S13/Tables 1 and S3	smaller sized particles are preferred because they allow for narrower bands and because their overall extinction mechanism favors absorbance rather than scattering
2. particle interaction with substrate	Figures 6 and S14	NP stabilizing environments should be chosen to allow minimal interaction with the substrate while also being permeable to the analyte of interest
3. specular vs diffuse	Figure 3/Table 1	particles should be selected to have a higher specular (or absorbance) component. Measurements should include either the specular or total spectra. In all cases, the measurement configuration and geometry should be reported
4. measurement angle	Figures 4, S10 and S11	measurements at low angles are preferred to allow the highest intensity measurements. The measurement angle should match the angle of incident light to allow collection of the specular component
5. surface roughness	Figures S15–S17/Table S5	surface roughness of the substrate has minimal influence on the spectral profiles. This allows unpolished substrates or irregular geometries to be used
6. particle deposition	Figure 2 and S3	dense NP depositions are preferred for high intensity, low broadening spectral measurements. Drop casting enables fast deposition of dense particles, but other techniques such as spray deposition, electrodeposition, and seed mediated electroless deposition may allow larger coverage while achieving more uniform coatings

### 4.1 Particle Choice

Plasmonic NPs that favor absorbance over scattering allow for simpler interpretation of reflectivity measurements. When absorbance is the main extinction mechanism, the observed reflectivity peaks of the NP on substrates were narrower and mainly specular in nature. This would be especially important for sensing mechanisms that rely on subtle shifts in the peak wavelength upon interaction with an analyte. Larger NPs tend to have high scattering components, as seen in the case with the 100 nm Au urchins.^16,45^ This resulted in broader spectra and large diffuse reflectivity contributions. For this reason, we recommend using smaller sized particles for sensing that favor absorbance as their main extinction mechanism. Finally, a consequence of these ensemble broadened measurements is the requirement of larger wavelength shifts for sensing. Plasmonic NPs that experience larger shifts upon exposure to their analyte of interest will be better suited for these types of applications.

### 4.2 Particle Interaction with the Substrate

In general, the particles on aluminum had smaller spectral shifts, and a smaller amount of peak broadening than those deposited on steel. However, certain stabilizing environments resulted in narrower and better-defined peaks on the steel substrates than others. CTAB particles tended to have very broad and poorly defined peaks, which make them undesirable candidates and could suggest that they interact more strongly with each other than with the substrates. However, the citrate and thiol stabilized particles showed narrow peaks on both the steel and aluminum substrates and the thiols showed smaller red shifts on the steel substrates compared to the other samples considered. When choosing a stabilizing environment for the NPs, careful consideration should be given to choosing one that is permeable to the analyte of interest but allows minimal interaction with the substrate. Additionally, an advantage that we observed to using the metal substrates was the lack of near field coupling between the particles, despite being arranged in cluster formation on the substrate. This feature can be seen as advantageous over other substrates, such as indium tin oxide and would be better able to be applied in real world sensing environments. To build on the empirical set of design rules presented here, future work could probe the influence that hydrophilic or hydrophobic ligand environments have on their interaction with the substrate by examining series of ligands of similar carbon chain lengths but varied functionalization to control the AuNP separation while tuning their hydrophilicity.

### 4.3 Specular Versus Diffuse Component

In general, the specular component had higher intensities and sharper peaks when compared to the diffuse component of the spectra. This was observed for the both the various capping agents, as well as the different AuNP shapes. Additionally, collection of the diffuse reflectance typically requires use of specialized equipment such as an integrating sphere, while the specular component can be collected with simpler configurations as long as the measurement angle is the same angle as the incident light or the specular exclusion port is closed when an integrating sphere is used. For these reasons, we recommend selecting samples that have higher specular (or absorbance) components and collecting either the specular or total spectra. In all measurements, the measurement configuration and geometry should be reported to facilitate reproducibility.

### 4.4 Measurement Angle

The measurement angle had a large influence on the overall intensity of reflectivity observed. Low measurement angles provided the best intensities and spectral resolution, with wide angles resulting in very low intensities. For sensing applications, control over measurement angle is crucial for measurements that rely on a change in signal intensity. The best readouts will be obtained at low angles relative to the normal of the substrate. Additionally, the limitations that variable angle measurements present in separation of specular from diffuse components further support the recommendation of using smaller particles that primarily have an absorbance component to their overall extinction. For this reason, the measurement angle should be at the same angle as the incident light, to allow collection of the specular component.

### 4.5 Surface Roughness

The surface roughness had minimal influence on adjusted reflectivity spectral profiles observed. Since uniform polishing of large substrates would be difficult to achieve, this shows the feasibility of scale up of LSPR based sensors on steel or aluminum substrates. This not only helps with cost of implementation by eliminating the requirement for time-consuming polishing steps but also allows for larger areas to be covered with nanoparticles to achieve higher signal intensities. Additionally, this suggests that substrates with irregular geometries that otherwise would be challenging to polish could be used, as long as they allow for low measurement angles as discussed previously in Section 4.4.

### 4.6 Particle Deposition

While the investigation of various NP deposition methods was outside the scope of the experiments presented in this work, one can assume that deposition technique will impact the overall signal intensity due to its influence on the uniformity and density of particles on the substrate. In general, more particles will allow more light to be absorbed, but if they are stacked on top of each other, there will be diminishing improvement in signal intensity. Typically, measurements that yielded higher intensities had a lower degree of peak broadening, so more uniform and dense NP depositions will allow improved spectral measurements. Additionally, uniformity from substrate to substrate is desired to allow better quality control between sensors and require less calibration. While the drop casting method used for this work was fast and simple, it suffers disadvantages in terms of scalability and having a coffee ring effect which causes nonuniformity of the arrangement of the AuNPs. Future work will explore alternative techniques to prepare nanoparticle coatings on surfaces. Methods such as spray deposition,^53^ electrodeposition,^54^ Langmuir–Blodgett films,^55^ and seed mediated electroless deposition^56^ have been applied to deposit plasmonic nanomaterials on substrates. These techniques allow larger areas to be covered while achieving more uniform coatings and control over film thickness. Further studies will explore these techniques to deposit AuNPs on steel and aluminum substrates and establish protocols to control the density and thickness of the coatings of the AuNPs could better optimize reflectivity measurements. The reflectivity intensity can then be correlated with AuNP density and thickness through image analysis to determine the ideal AuNP coating for optimized signal readout. While future work can expand our understanding of the influence of particle deposition on the substrates, the findings in this work suggest design rules even with nonuniform AuNP depositions.

## 5 Conclusions

In conclusion, we deposited AuNPs with varying stabilizing environments on nonpristine aluminum and steel substrates and measured their reflectivity spectra. We considered various parameters, including AuNP size and shape, measurement angle, and varying degrees of roughness of the surface and determined which factors had the most and least impact on the observed spectra. Based on our observations, we have outlined a series of guidelines that can be used when implementing plasmonic NPs into application including LSPR based sensing, SERS, or plasmon assisted catalysis. Future studies will consider additional factors such as hydrophilicity of the ligand environment, surface coatings on the substrates, and alternative approaches to depositing the AuNPs on the surface to better control the overall density and uniformity of the AuNPs. Additionally, the findings of this work can be applied to NPs that are sensitive to various analytes of interest to assess how well they respond when deposited on aluminum and steel substrates. This empirical work complements previous studies on pristine surfaces and provides a new insight for the simple implementation of AuNPs on industrially relevant substrates without requiring highly ordered or pristine surfaces or complicated fabrication methods.

## Supplementary Material



## Data Availability

All data can be found within the manuscript and Supporting Information.

## Notes

DLS data, SEM images and image analysis details, details on treatment of reflectivity data, and raw spectra for all reflectivity measurements (PDF)

## #

Donald P. Shiley School of Engineering, University of Portland, Portland, OR 97203, United States
