# Supplementary material for: Investigation of the Nanoparticle–Support Interaction Effects of Plasmonic Gold Nanoparticles Deposited on Nonpristine Aluminum and Steel Substrates
Source: ACS Omega. 2026 Sep 23;11(39):58520–31. doi: 10.1021/acsomega.5c11527 (PMC13647390; doi:10.1021/acsomega.5c11527)
Supplement: Supplementary file 1 [file ao5c11527_si_001.pdf]

Supporting Information:

Investigation of the nanoparticle – support interaction effects of plasmonic gold nanoparticles deposited on non-pristine aluminum and steel substrates.

Rosemary L. Calabro [a, c], F. John Burpo [a, b], Stephen F. Bartolucci [c]*, Joshua A. Maurer [c]*

a. Department of Chemistry and Life Science, United States Military Academy, West Point, NY 10996, USA.
b. Photonics Research Center, United States Military Academy, West Point, NY 10996, USA.
c. U.S. Army Combat Capabilities Development Command-Armaments Center, Watervliet Arsenal, NY 12189, USA.

*Corresponding author

Email: joshua.a.maurer4.civ@army.mil

Keywords: Plasmonics, nanoparticles, LSPR, gold nanoparticles, reflectivity

Characterization of the 50 nm AuNPs

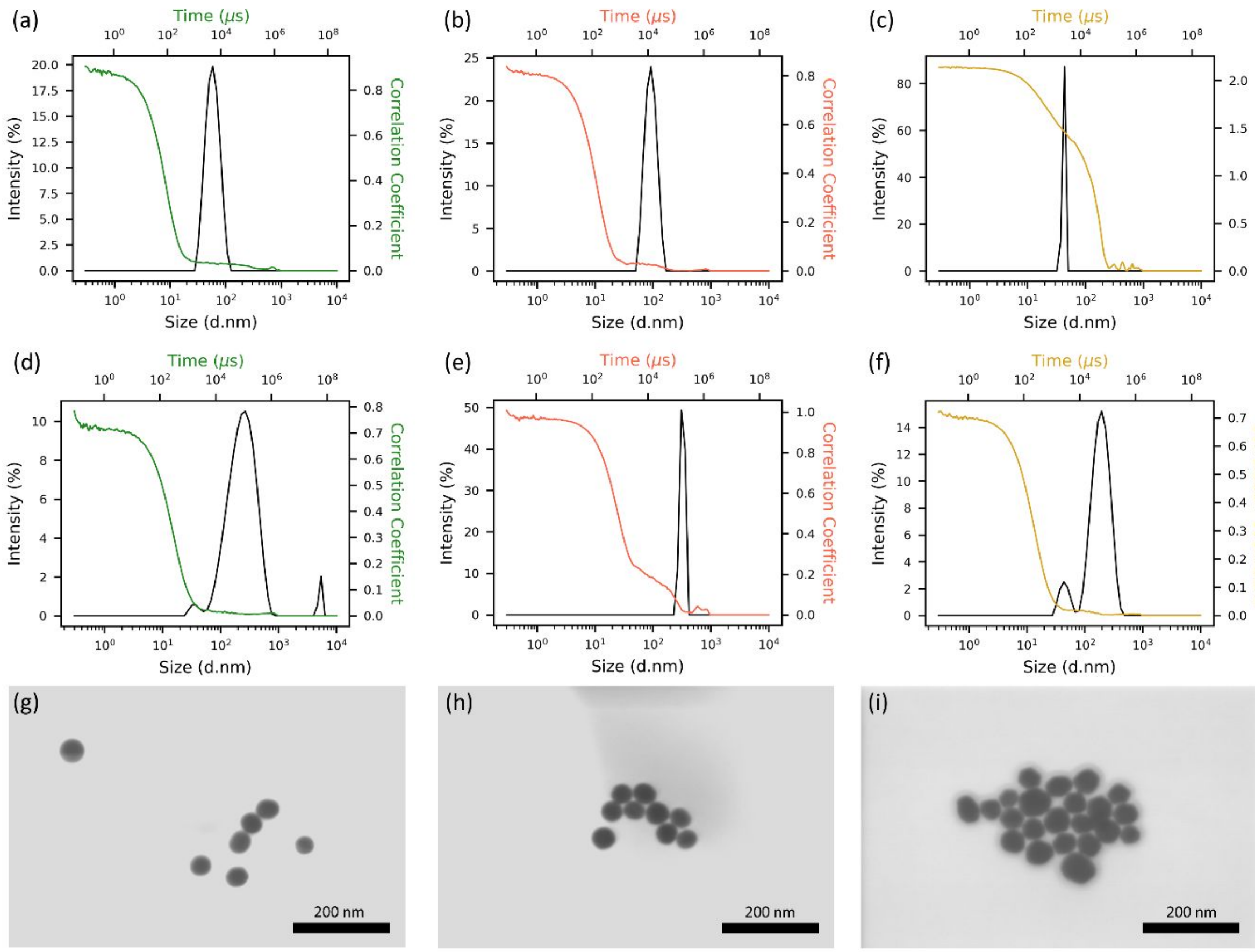

Figure S1. DLS of 50 nm AuNPs in water stabilized with citrate (a), CTAB (b), and phosphate buffered saline (c), DLS of 50 nm AuNPs in IPA stabilized with citrate (d), CTAB (e), and phosphate buffered saline (f), and STEM images of the 50 AuNPs stabilized by citrate (g), CTAB (h), and phosphate buffered saline (i).

ImageJ analysis

The freely available ImageJ software package[1] was used to perform cluster analysis of the AuNPs on the steel and aluminum substrates. For each substrate, 6 SEM images of the AuNPs deposited on the substrate at 20,000× magnification were analyzed. Figure S2a shows a flowchart of the general image analysis procedure. For each image, the raw image (Figure S2b) was converted to a binary (Figure S2c) where the dark regions corresponded to AuNPs. The binaries were carefully inspected to ensure no artifacts were included as dark spots and they were removed using the “fill flood” tool if necessary. The binary was then analyzed using the “analyze particles” tool using a threshold of 0.0005 μm^2 to infinity for size and excluding on edges. This generated an overlay mask (Figure S2d) where each individually colored region is numbered and corresponds to a calculated area. The areas were sorted into two groups. All areas < 0.004 μm^2 were characterized as individual particles and all areas ≥ 0.004 μm^2 were characterized as clusters containing two or more AuNPs based on the calculated area of the 50 nm AuNPs and visual inspection of several particles and clusters to confirm the threshold area for individual particles. To determine the number of particles in each cluster, the cluster areas were divided by the average area of the individual particles and rounded to the nearest integer value.

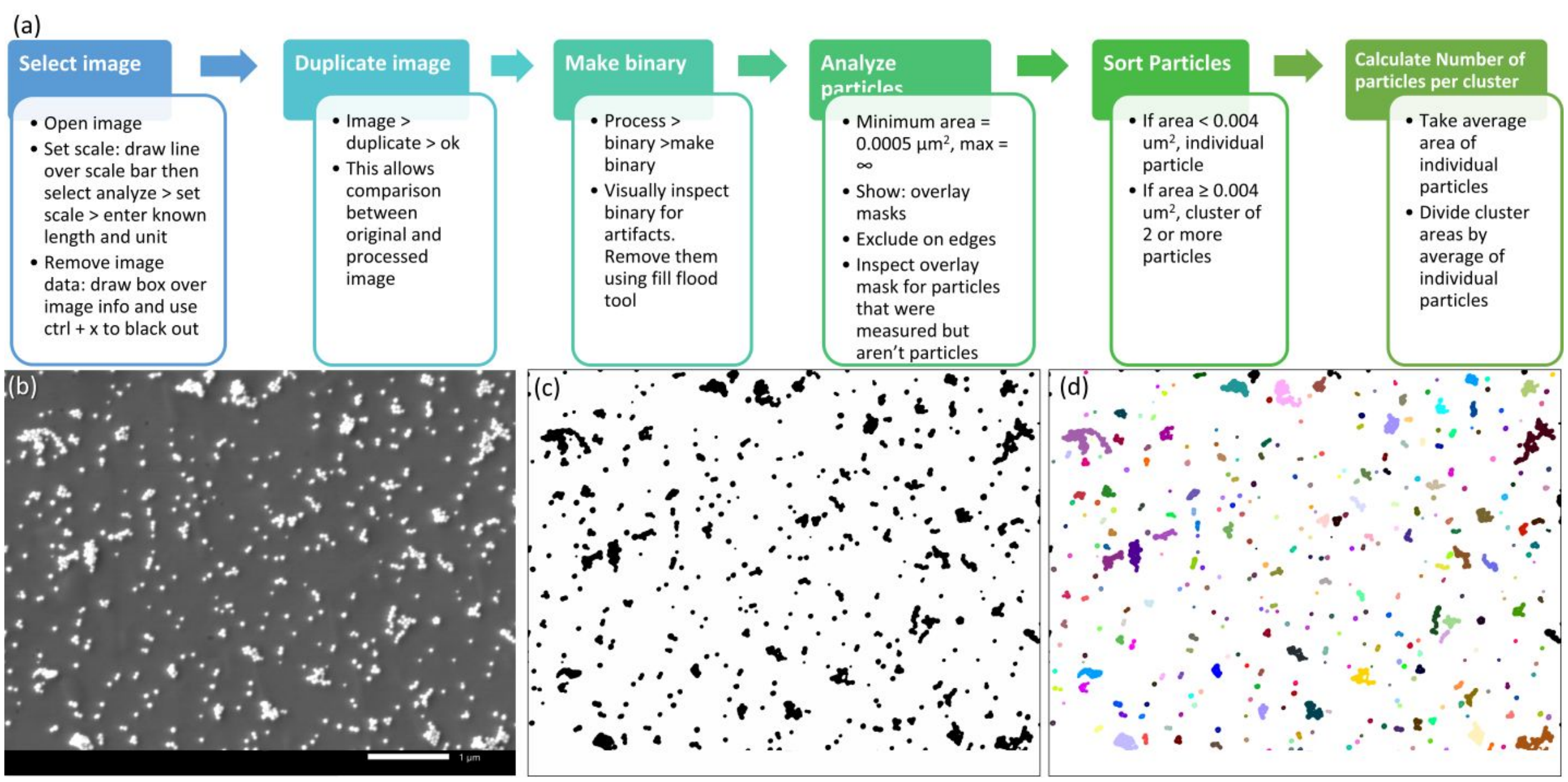

Figure S2. Flowchart of the image analysis procedure (a), and a sample SEM image of AuNPs on steel throughout the ImageJ cluster analysis process showing the raw image (b), the image after making a binary (c), and the image after applying particle size analysis (d). Each colored region is numbered and corresponds to either an individual or cluster of AuNPs.

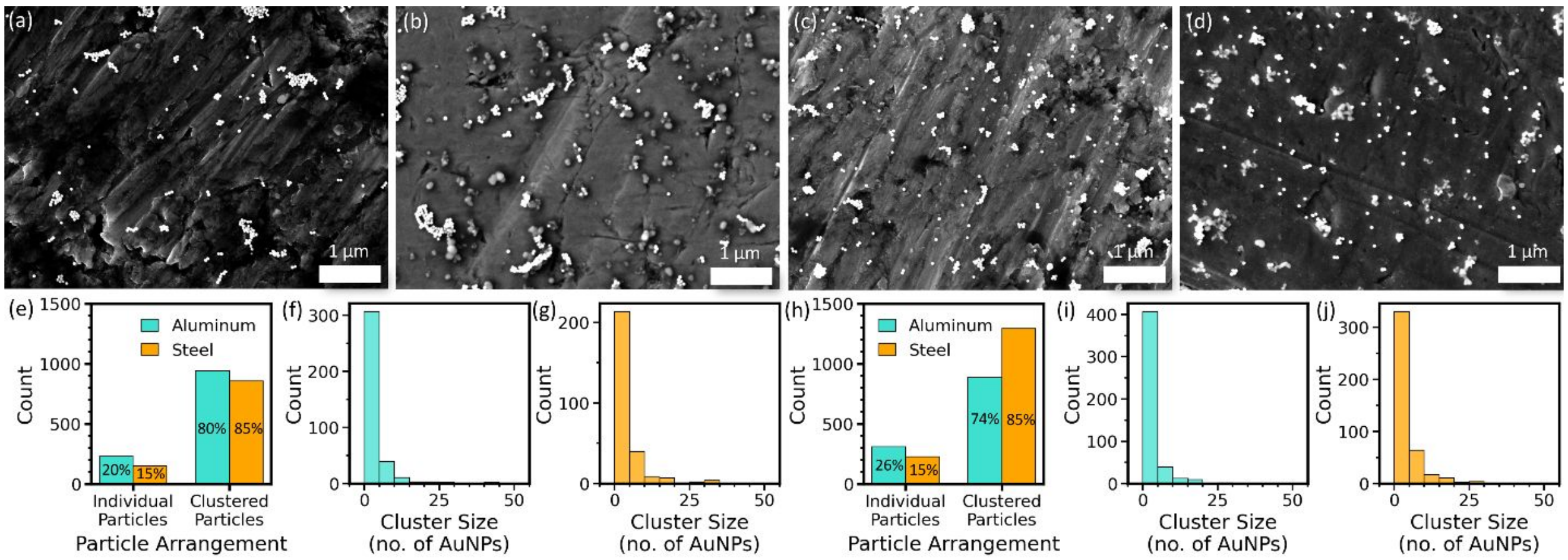

Figure S3. SEM images of the citrate stabilized (a-b) and CTAB stabilized (c-d) AuNPs deposited on aluminum (a, c) and steel (b, d), box plots summarizing the cluster analysis of the citrate (e) and CTAB (h) stabilized AuNPs, and histograms of the citrate (f, g) and CTAB (i, j) stabilized particles on aluminum (f, i), and steel (g, j).

Treatment of AuNP on Substrate Data

For polished substrates, the primary component of total reflected light will be specularly reflected light which is the light reflected at the same angle as the incident light. The diffusely reflected light, which includes the light reflected in all directions, is also included in the measurement but has a significantly lower contribution as shown in Figure S4 for both the aluminum and steel substrates. Figure S5 presents schemes of the various possibilities of the substrates and AuNPs interacting with incident light when AuNPs are deposited on the substrates. The first option is that the AuNP specularly reflects or transmits the incident light. This would result in no observed change in the %R since the same amount of light is reflected back to the detector when compared to the bare substrate. The next option is that the AuNP absorbs the incident light. This would result in a decrease in the %R at wavelengths where the AuNP absorbs relative to the specular reflectance of the bare substrate. Example spectra of this are shown in panels b and d of Figure 2 in the main text and panels a and c of Figures S6 and S7. A pseudo absorbance spectrum can be obtained by calculating the change in reflectivity, Δ%R, using the following equation:

$$\Delta\%R = \%R_{\text{substrate , specular}} - \%R_{\text{AuNP, specular}} \qquad \text{Equation (1)}$$

The key factor in this calculation is that the measurements of both the bare substrate and the substrate with the AuNPs were collected with the specular exclusion port closed. The third option is that the AuNPs diffusely reflect the incident light, which occurs when the AuNPs have a scattering component in the overall extinction. Unlike the case where the specular components are included, this scenario results in an increase in the overall %R since it causes more light to be reflected to the detector compared to the bare substrate. For this measurement, the spectra are collected with the specular exclusion port open. As with option 2, a pseudo absorbance spectrum can be obtained, however, in this case the bare substrate is subtracted from the substrate with AuNPs as shown in the following equation:

$$\Delta\%R = \%R_{AuNP\,,\,diffuse} - \%R_{substrate,\,diffuse} \qquad \text{Equation (2)}$$

Finally, a slight overall decrease in intensity occurs due to impurities in the IPA solvent used to dropcast the AuNPs as shown in Figure S8. To account for this, the Δ%R spectra are also offset to zero prior to plotting. The final plots were termed "adjusted spectra" to indicate that they were background subtracted and offset to zero. To calculate the peak broadness of the substrate measurements, the intensity of the peak maximum was estimated from the adjusted spectra. The wavelength at which half of the intensity was observed was measured from the longer wavelength portion of the spectra. The half width half maximum was then calculated by subtracting the two wavelengths. These calculated peak broadness values are reported in Tables S3-S4.

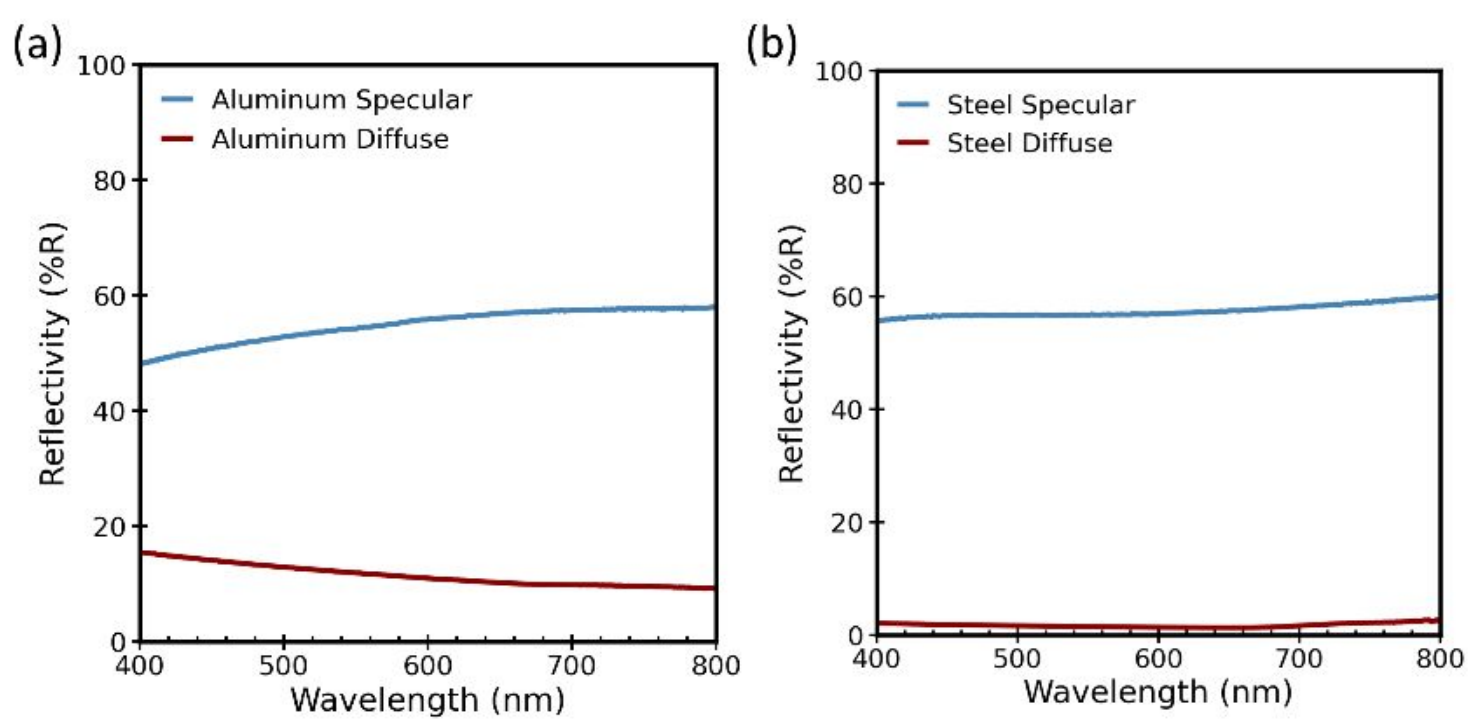

Figure S4. Specular and diffuse reflectivity measurements of a final polished aluminum (a) and steel (b) substrate.

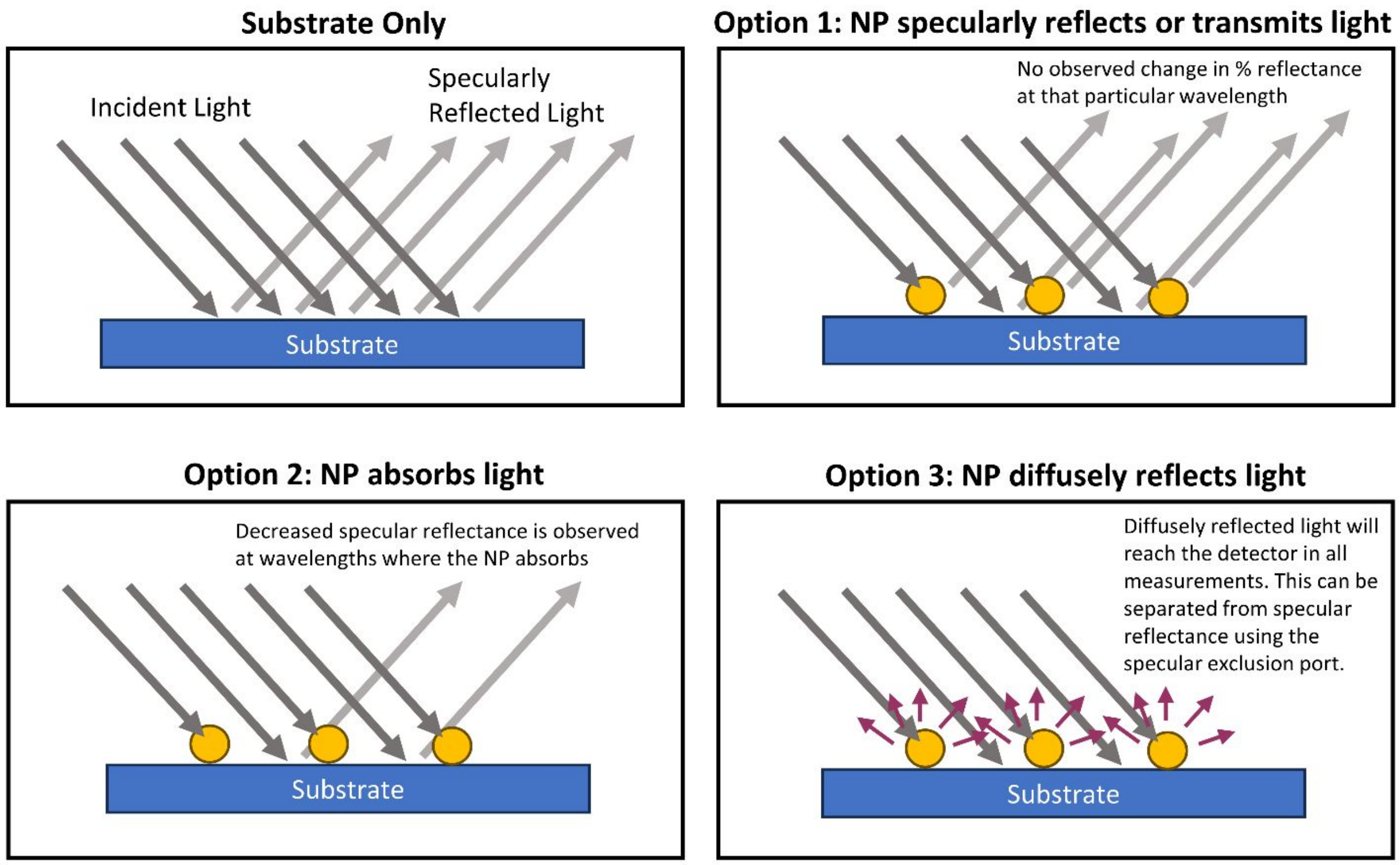

Figure S5. Scheme of the various possibilities for light interacting with the AuNPs deposited on polished substrates.

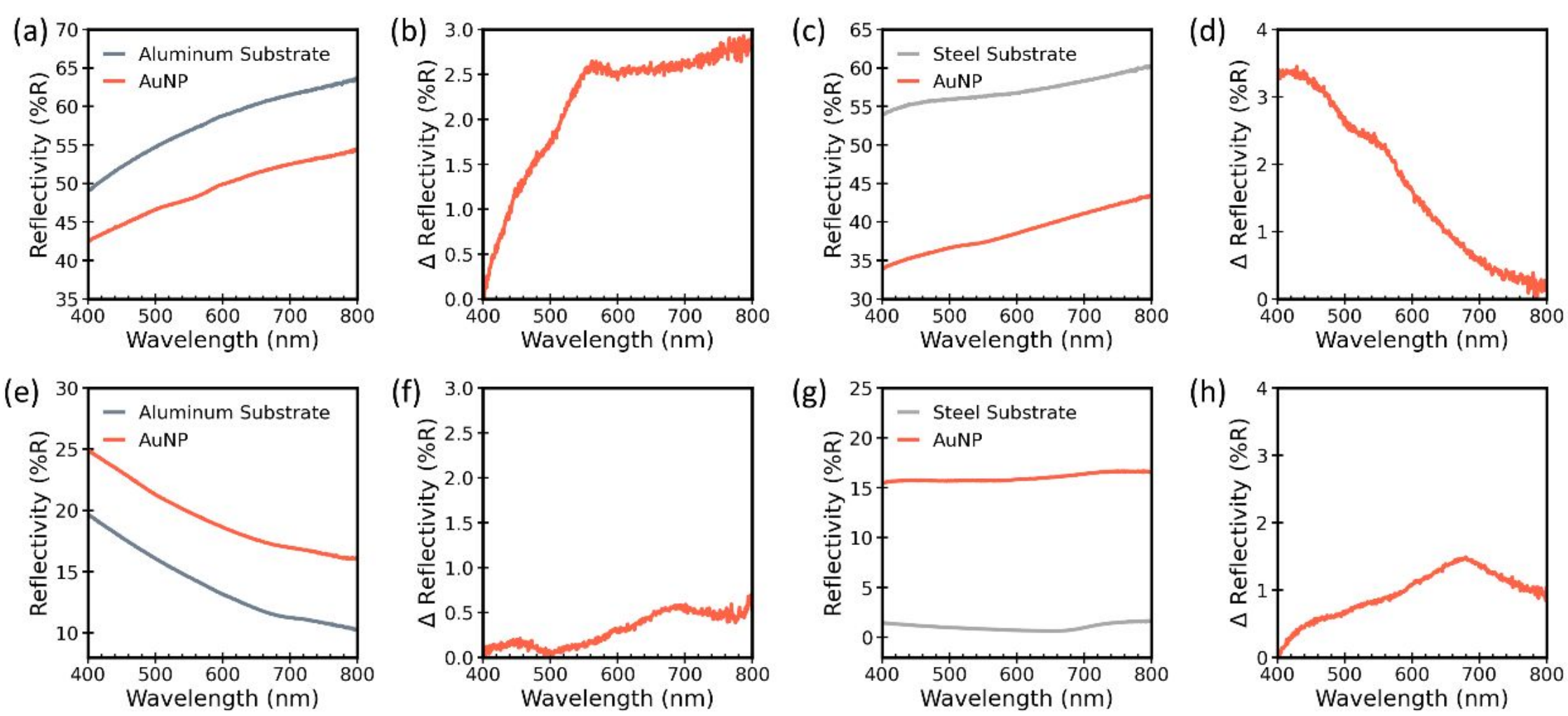

Figure S6. Specular and diffuse raw and adjusted reflectivity spectra of CTAB coated AuNPs. Specular on aluminum (a, b), specular on steel (c, d), diffuse on aluminum (e, f), and diffuse on steel (g, h).

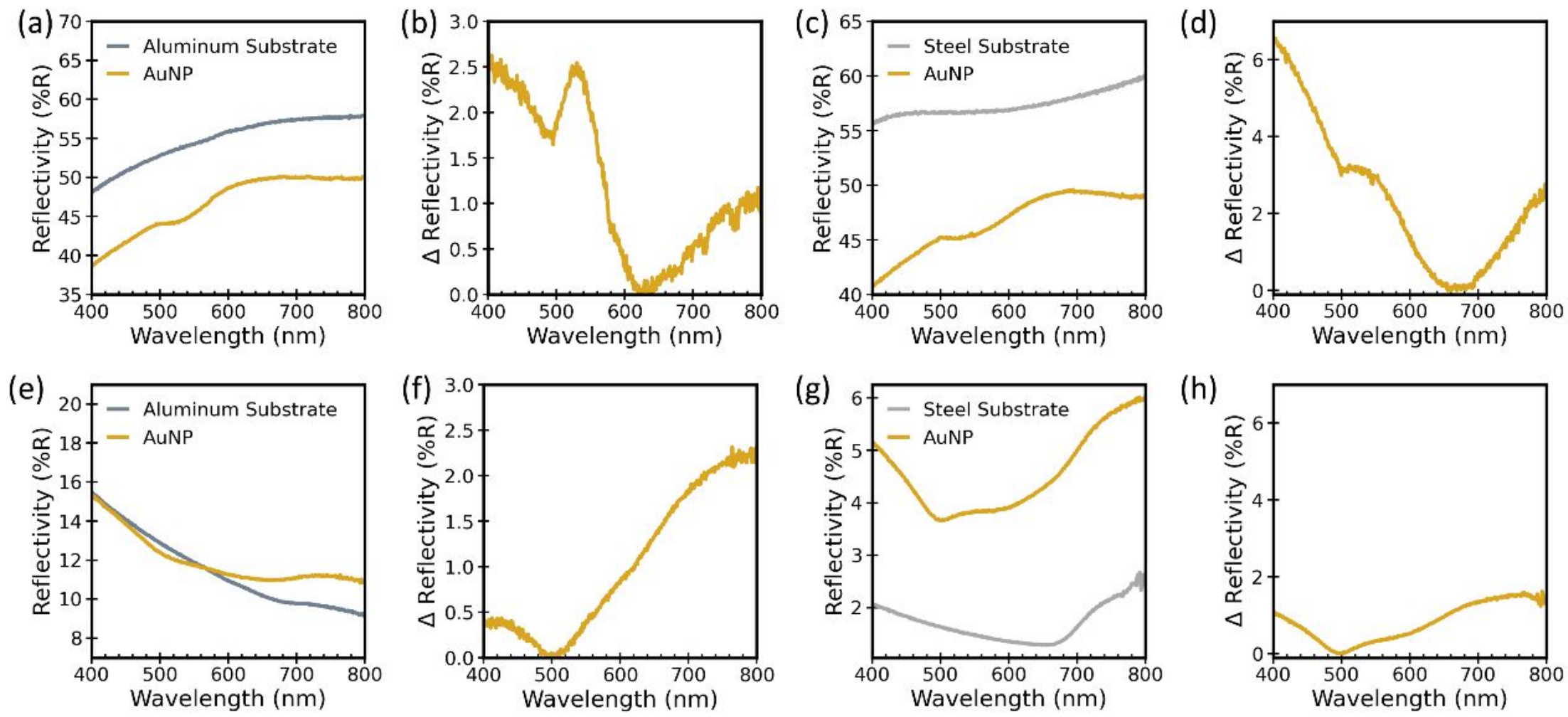

Figure S7. Specular and diffuse raw and adjusted reflectivity spectra of phosphate buffered saline stabilized AuNPs. Specular on aluminum (a, b), specular on steel (c, d), diffuse on aluminum (e, f), and diffuse on steel (g, h).

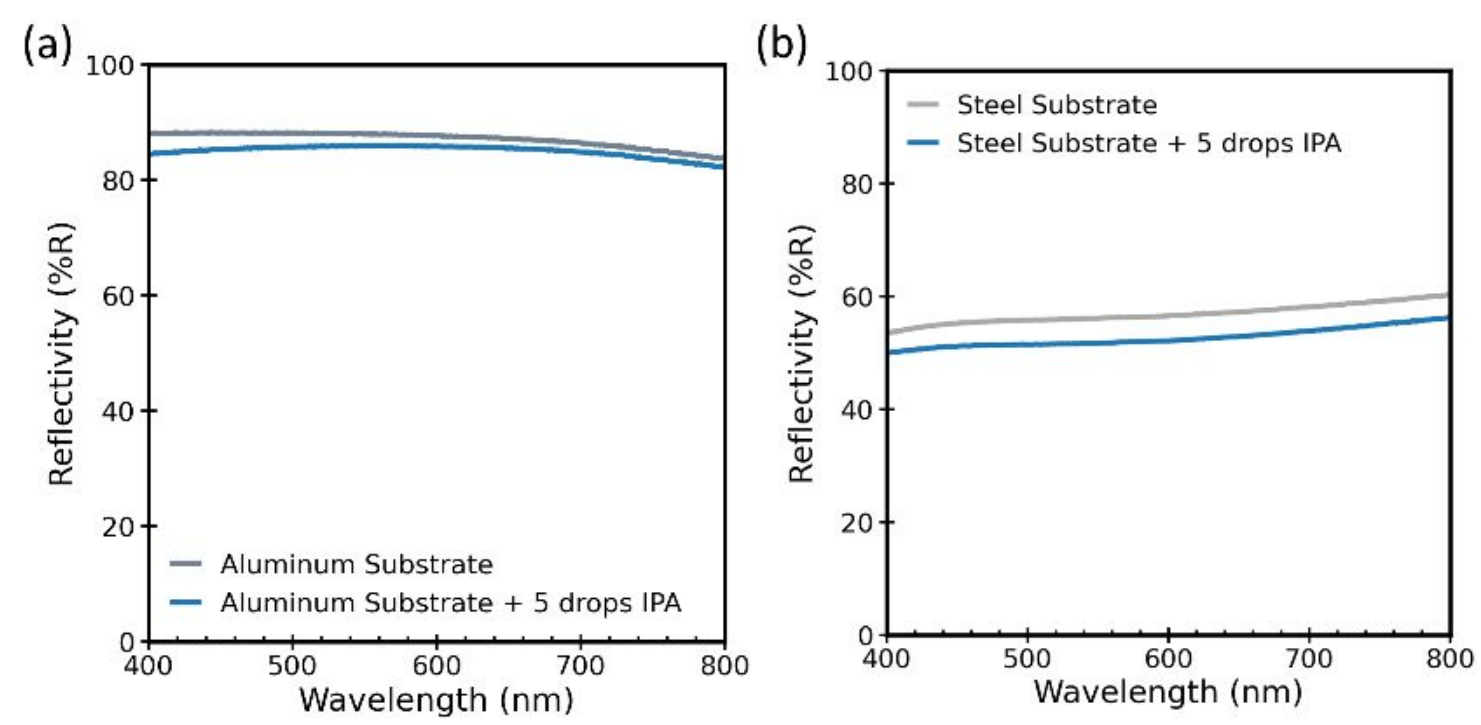

Figure S8. Reflectivity spectra of aluminum (a) and steel (b) substrate before and after adding dropcasting IPA.

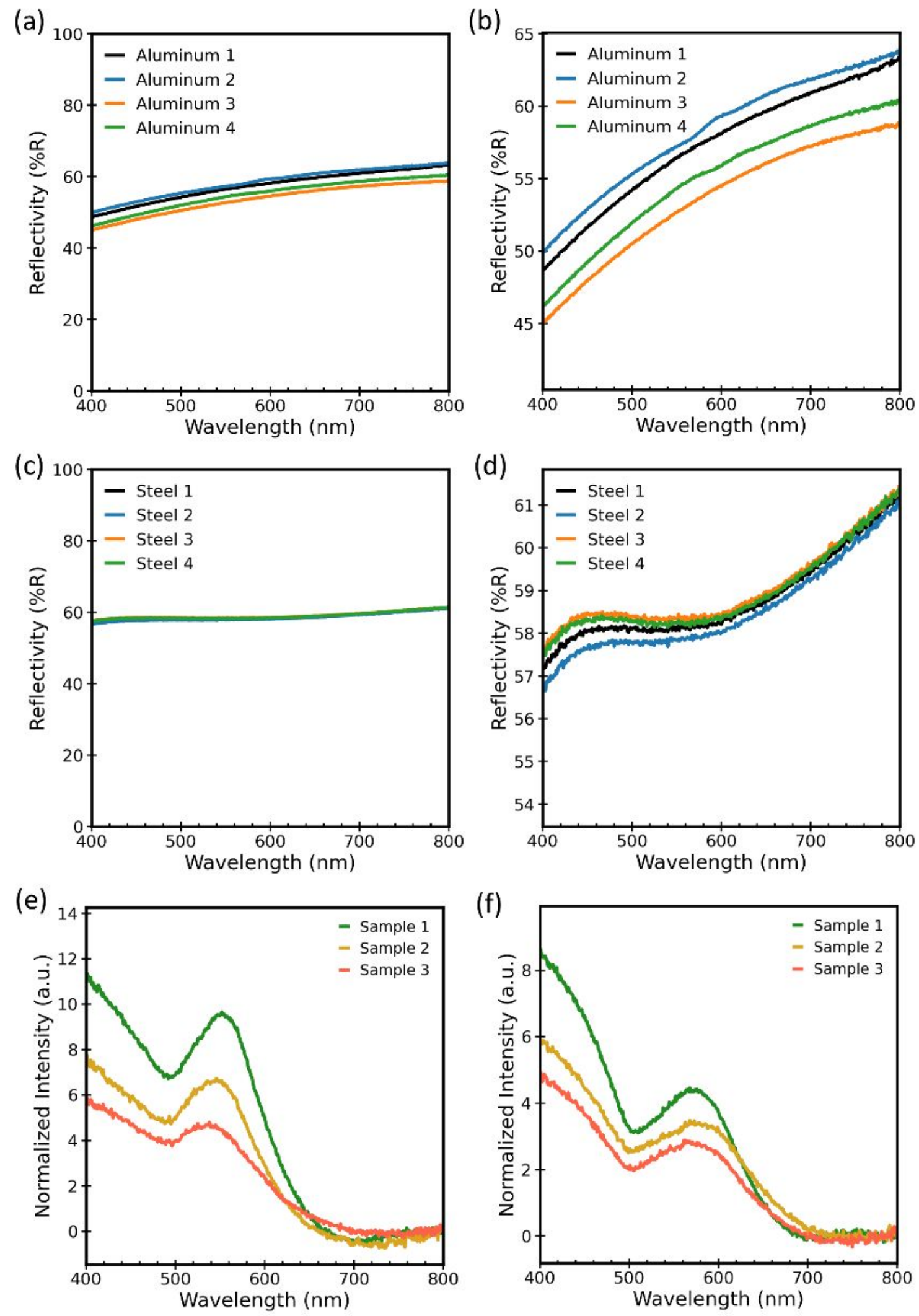

Figure S9. Specular reflectivity measurements of several aluminum (a, b) and steel (c, d) final polished substrates indicate reproducibility of the polishing process. Multiple repetitions of the citrated coated AuNPs drop cast on final polished aluminum (e) and steel (f) show some variation in intensity from sample to so sample but overall similar spectral profiles.

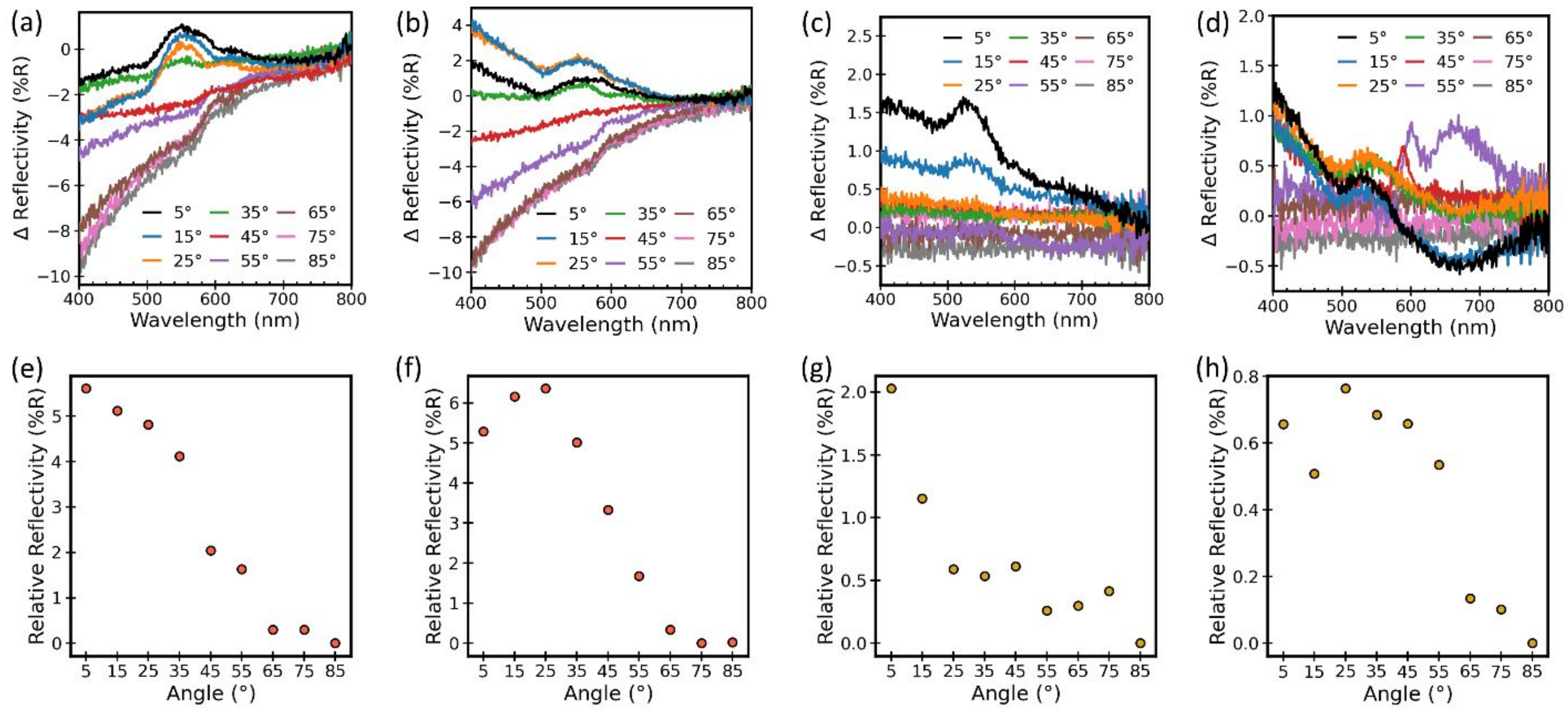

Figure S10. Variable angle measurements and scatter plots of the relative reflectivity at various measurement angles for the CTAB coated AuNPs deposited on aluminum (a, e) and steel (b, f), and the phosphate buffered saline stabilized AuNPs deposited on aluminum (c, g), and steel (d, h).

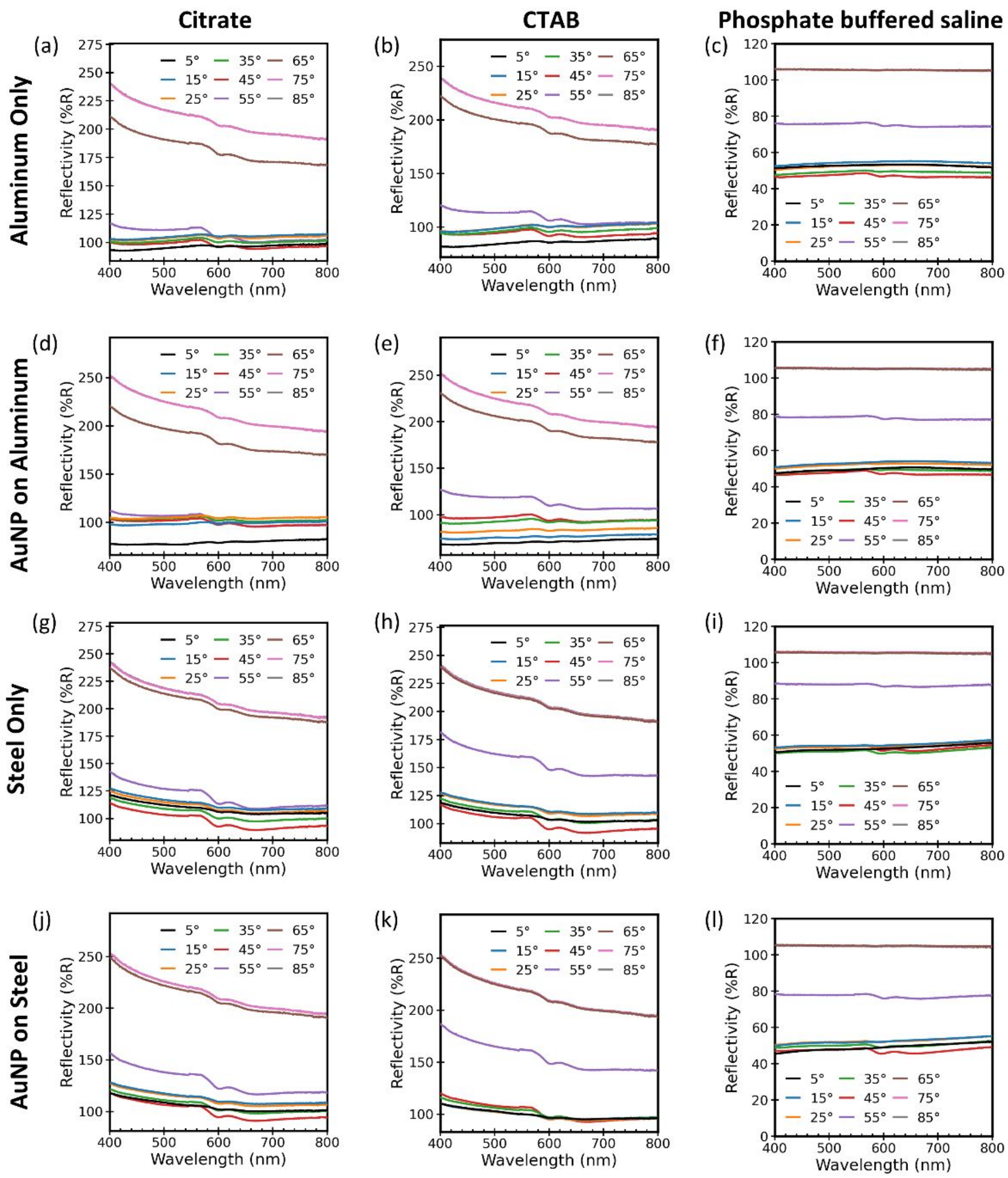

Figure S11. Raw variable angle data for the aluminum substrates used for citrate (a), CTAB (b), and phosphate buffered saline (c) stabilized particles, the AuNPs deposited on the aluminum substrates and stabilized with citrate (d), CTAB (e), and phosphate buffered saline (f), the steel substrates used for citrate (g), CTAB (h), and phosphate buffered saline (i) stabilized particles, and the AuNPs deposited on the steel substrates and stabilized by citrate (j), CTAB (k), and phosphate buffered saline (l).

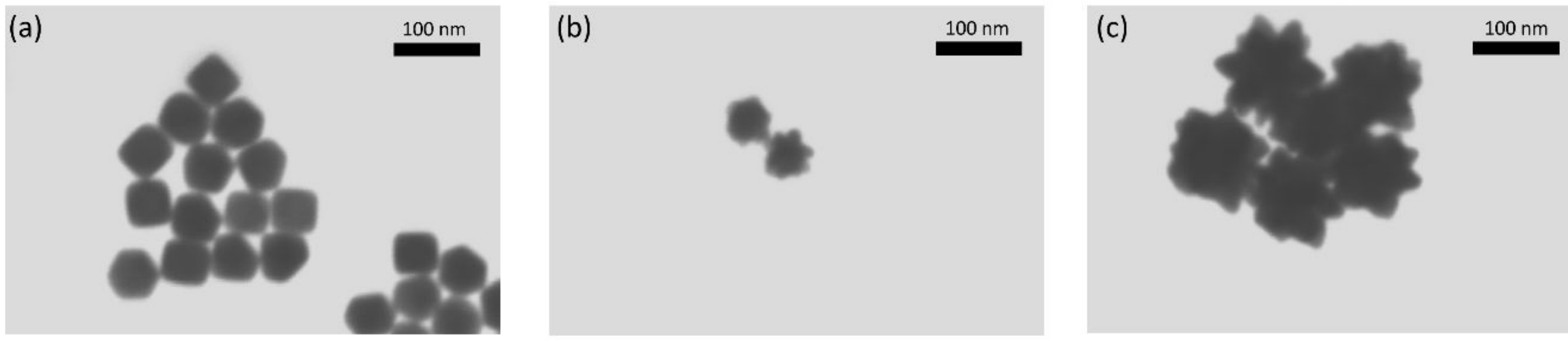

Figure S12. SEM images of the 50 nm Au cubes (a) 50 nm Au urchins (b), and 100 nm Au urchins (c).

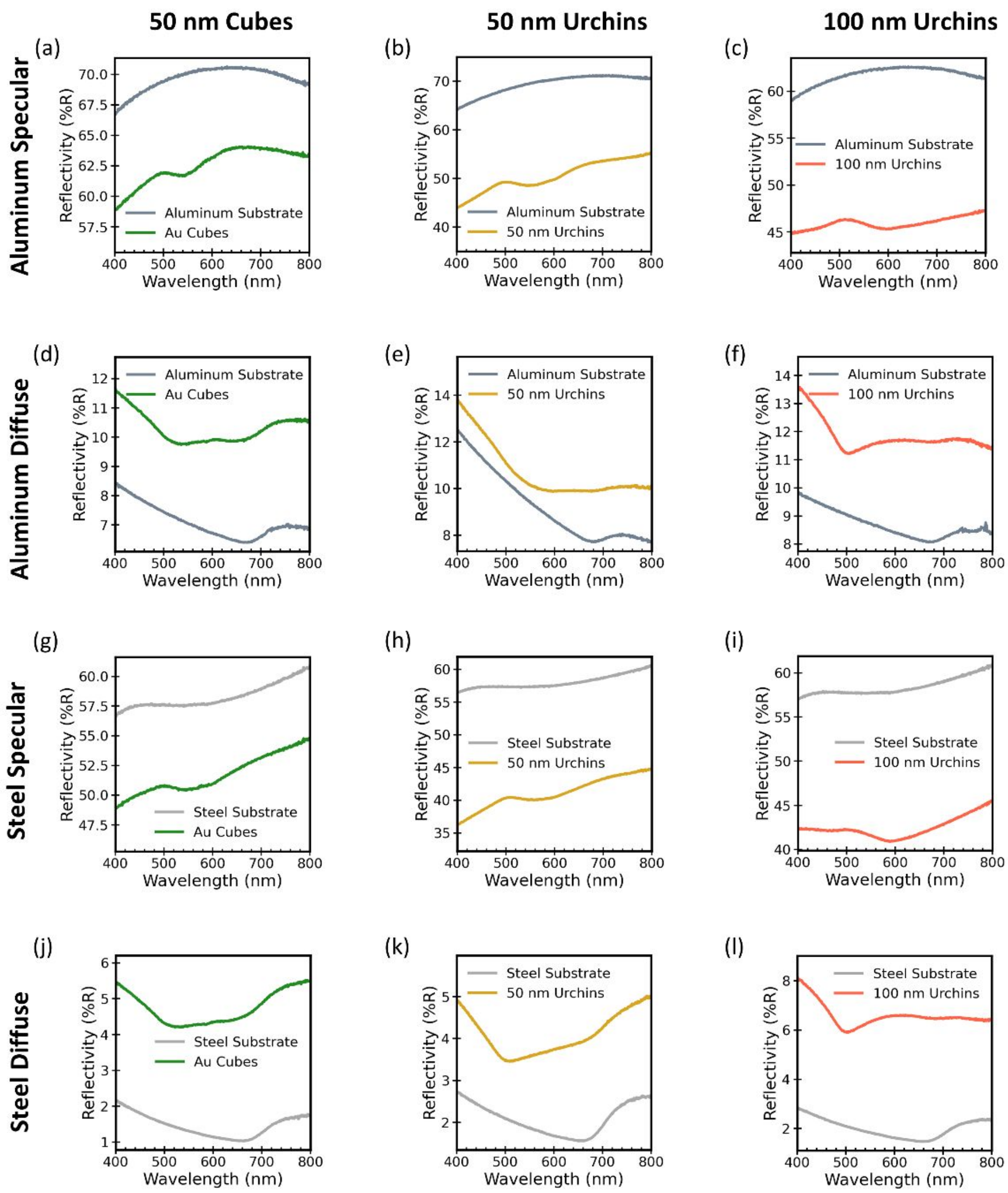

Figure S13. Specular and diffuse raw spectra for 50 nm Au cubes on aluminum (a, d) and steel (g, j), the 50 nm Au urchins on aluminum (b, e) and steel (h, k), and the 100 nm Au urchins on aluminum (c, f), and steel (i, l).

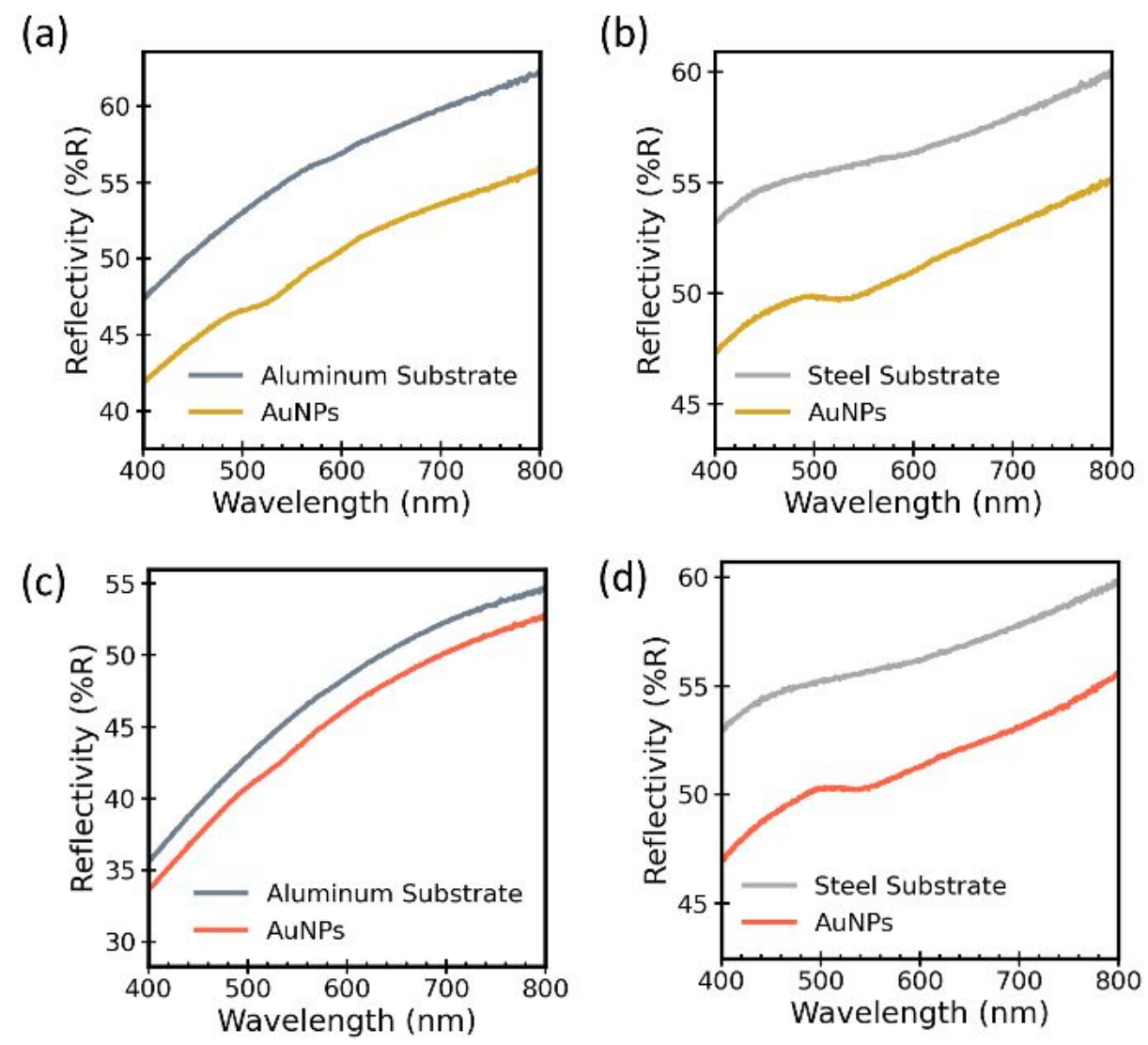

Figure S14. Raw reflectivity spectra for ligand exchange AuNPs coated with 1-propanethiol and deposited on aluminum (a) and steel (b) and the AuNPs coated with 1-hexadecanethiol and deposited on aluminum (c) and steel (d).

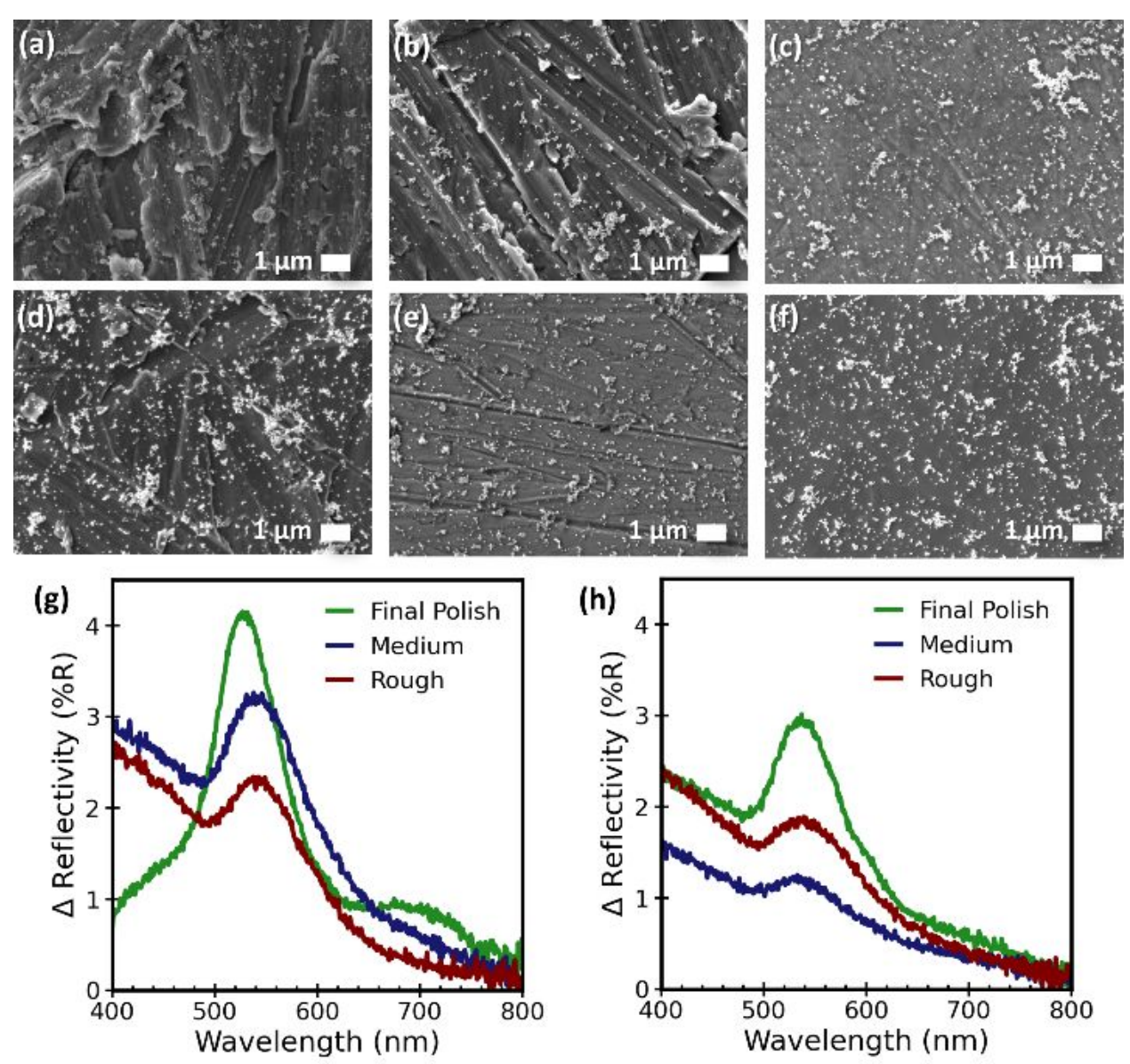

Figure S15. AuNPs deposited on an aluminum substrate with a rough (a), medium (b), and final polish (c), and on a steel substrate with a rough (d), medium (e), and a final polish (f), and the reflectivity spectra of the citrate stabilized AuNPs deposited on the aluminum (g) and steel (h) substrates.

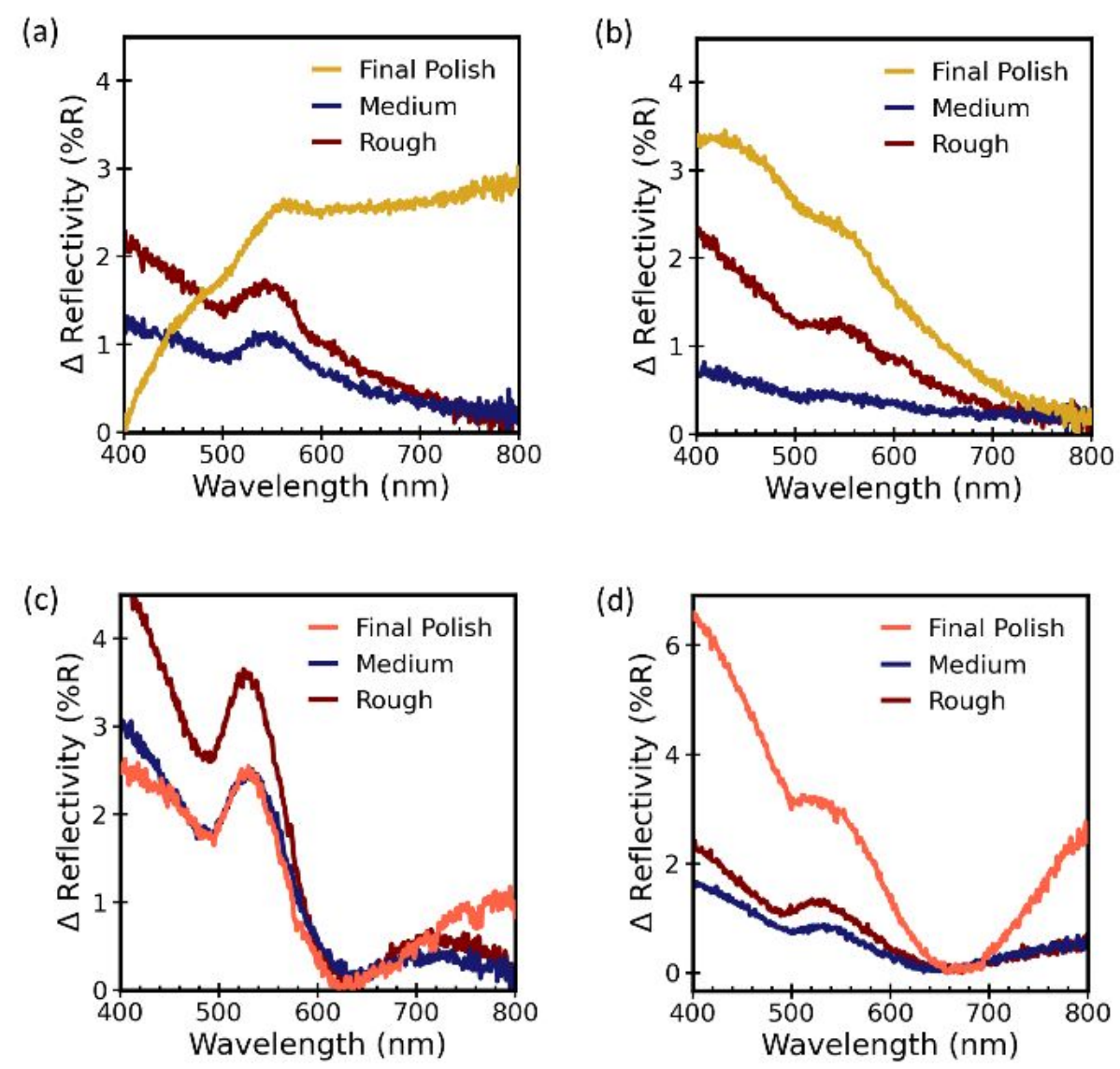

Figure S16. Surface roughness measurements of the CTAB stabilized AuNPs deposited on aluminum (a) and steel (b) and the phosphate buffered saline stabilized AuNPs deposited on aluminum (c) and steel (d).

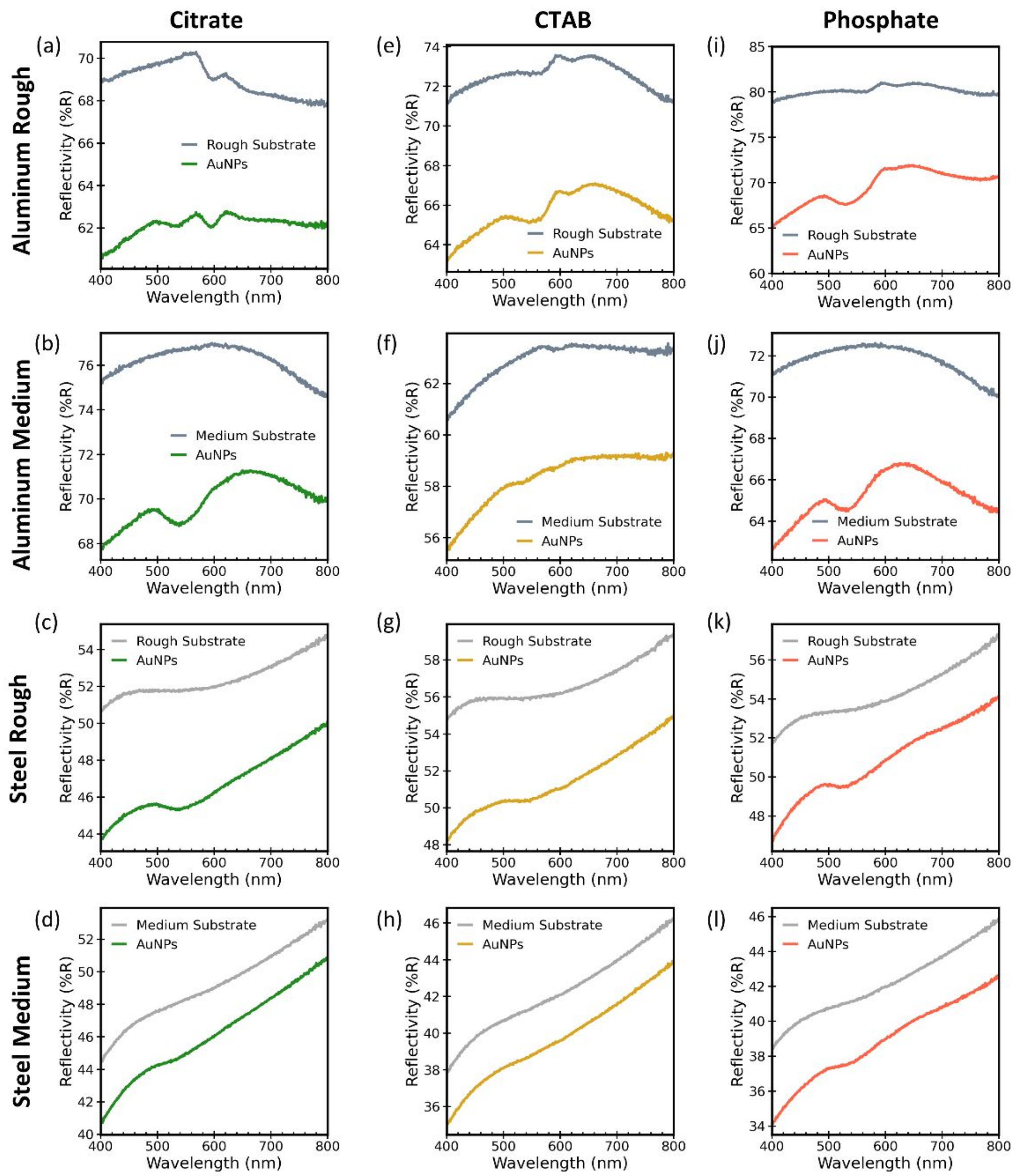

Figure S17. Raw reflectivity spectra for 50 nm AuNP citrate stabilized AuNPs on rough (a) and medium (b) polished aluminum and rough (c) and medium (d) polished steel, the CTAB stabilized AuNPs on rough (e) and medium (f) polished aluminum and rough (g) and medium (h) polished steel, and the phosphate buffered saline stabilized AuNPs on rough (i) and medium (j) polished aluminum and rough (k) and medium (l) polished steel.

Table S1. DLS peak Intensities of the 50 nm AuNPs with various stabilizing environments

Sample	Polydispersity Index (PI)	Peak One Mean by Intensity (nm)	Peak One Area by Intensity (%)	Peak Two Mean by Intensity (nm)	Peak Two Area by Intensity (%)
Citrate AuNPs in H_2O	0.1648	60.22	100	-	-
Citrate AuNPs in IPA	0.4075	259.8	95.48	5309	2.549
CTAB AuNPs in H_2O	0.2925	94.2	100	-	-
CTAB AuNPs in IPA	0.5525	326.1	100	-	-
Phosphate AuNPs in H_2O	1.951	42.9	100	-	-
Phosphate AuNPs in IPA	0.3611	200.4	91.28	45.57	8.724

Table S2. Solution phase spectral peak positions of the various 50 nm AuNPs, and extinction (Ext), absorbance (Abs), and scattering (Sca) peak wavelengths from true absorbance measurements.

Sample	Peak position in H_2O (nm)	Peak 1 position in IPA (nm)	Peak 2 position in IPA (nm)	Ext (nm)	Abs (nm)	Sca (nm)
Citrate AuNPs	531	539	685	532	530	567
CTAB AuNPs	533	546	660	530	524	588
Phosphate AuNPs	527	542	702	528	524	542

Table S3. Spectral positions and estimated peak broadness of various AuNPs in solution and on aluminum and steel substrates.

	Solution (H_2O)		Aluminum Substrate		Steel Substrate	
Sample	Peak Wavelength (nm)	Peak Broadness (nm)	Peak Wavelength (nm)	Peak Broadness (nm)	Peak Wavelength (nm)	Peak Broadness (nm)
Citrate AuNPs	527	30	527	50	537	59
CTAB AuNPs	533	48	558	Broad Features	560	Broad Features
Phosphate AuNPs	531	36	530	38	541	53
100 nm Urchins	655	85	605	161	600	151
50 nm Urchins	561	48	566	76	560	78
50 nm Cubes	539	36	546	68	544	78

Table S4. Comparison of the peak broadness and peak intensity for AuNPs deposited on aluminum and steel substrates of varying degrees of polishing.

**Medium**	**Aluminum Substrate**			**Steel Substrate**		
Sample	Peak Wavelength (nm)	Peak Broadness (nm)	Peak Intensity (Δ%R)	Peak Wavelength (nm)	Peak Broadness (nm)	Peak Intensity (Δ%R)
Citrate AuNPs Repetition 1	552	48	9.7	568	55	4.5
Citrate AuNPs Repetition 2	546	48	6.7	568	70	3.5
Citrate AuNPs Repetition 3	538	58	4.8	563	68	2.9
Citrate AuNPs Final Polish	527	50	4.1	537	59	3
Citrate AuNPs Medium Polish	538	71	3.3	533	81	1.3
Citrate AuNPs Rough Polish	538	69	2.3	539	77	1.9
Phosphate AuNPs Final Polish	530	38	2.5	541	53	3.1
Phosphate AuNPs Medium Polish	532	54	3.3	532	55	2.2
Phosphate AuNPs Rough Polish	531	52	3.1	530	63	3

Table S5. Arithmetic mean roughness (Ra) of the various substrates

**Substrate**	**Ra (μm)**
Aluminum Final Polish	0.31
Aluminum Medium Polish	0.43
Aluminum Rough Polish	0.62
Steel Final Polish	0.33
Steel Medium Polish	0.39
Steel Rough Polish	0.60

Supporting References

(1) Schneider, C. A.; Rasband, W. S.; Eliceiri, K. W. NIH Image to ImageJ: 25 Years of Image Analysis. *Nature Methods* **2012**, *9* (7), 671–675. https://doi.org/10.1038/nmeth.2089.
